# Single cell RNA sequencing confirms retinal microglia activation associated with early onset retinal degeneration

**DOI:** 10.1038/s41598-022-19351-w

**Published:** 2022-09-10

**Authors:** Asha Kumari, Raul Ayala-Ramirez, Juan Carlos Zenteno, Kristyn Huffman, Roman Sasik, Radha Ayyagari, Shyamanga Borooah

**Affiliations:** 1 Shiley Eye Institute, University of California, San Diego, 9415 Campus Point Drive, La Jolla, CA 92093 USA; 2 Department of Biochemistry, Faculty of Medicine, UNAM, Mexico City, Mexico; 3 Department of Genetics, Conde de Valenciana, Institute of Ophthalmology, Mexico City, Mexico; 4 School of Medicine, Center for Computational Biology and Bioinformatics, University of California, San Diego, La Jolla, CA USA

**Keywords:** Hereditary eye disease, Retinal diseases

## Abstract

Mutations in the *Membrane-type frizzled related protein* (*Mfrp)* gene results in an early-onset retinal degeneration associated with retinitis pigmentosa, microphthalmia, optic disc drusen and foveal schisis. In the current study, a previously characterized mouse model of human retinal degeneration carrying homozygous c.498_499insC mutations in *Mfrp* (*Mfrp*^KI/KI^) was used. Patients carrying this mutation have retinal degeneration at an early age. The model demonstrates subretinal deposits and develops early-onset photoreceptor degeneration. We observed large subretinal deposits in *Mfrp*^KI/KI^ mice which were strongly CD68 positive and co-localized with autofluorescent spots. Single cell RNA sequencing of *Mfrp*^KI/KI^ mice retinal microglia showed a significantly higher number of pan-macrophage marker *Iba-1 and F4/80* positive cells with increased expression of activation marker (*CD68)* and lowered microglial homeostatic markers (*TMEM119, P2ry13, P2ry13, Siglech*) compared with wild type mice confirming microglial activation as observed in retinal immunostaining showing microglia activation in subretinal region. Trajectory analysis identified a small cluster of microglial cells with activation transcriptomic signatures that could represent a subretinal microglia population in *Mfrp*^KI/KI^ mice expressing higher levels of APOE. We validated these findings using immunofluorescence staining of retinal cryosections and found a significantly higher number of subretinal Iba-1/ApoE positive microglia in *Mfrp*^KI/KI^ mice with some subretinal microglia also expressing lowered levels of microglial homeostatic marker *TMEM119*, confirming microglial origin. In summary, we confirm that *Mfrp*^KI/KI^ mice carrying the c.498_499insC mutation had a significantly higher population of activated microglia in their retina with distinct subsets of subretinal microglia. Further, studies are required to confirm whether the association of increased subretinal microglia in MfrpKI/KI mice are causal in degeneration.

## Introduction

Microglia, are the primary immune cell of the retina and central nervous system (CNS)^1^. In healthy retina, microglia are primarily located in the inner plexiform layer (IPL) and the outer plexiform layer (OPL) as well as in ganglion cell layer (GCL) where they assist retinal homeostasis and immune surveillance. Changes in the retinal microenvironment can induce rapid microglial activation with release of pro-inflammatory cytokines, neurotoxic molecules, complement proteins and free radicals such as oxygen free radicals (ROS)^2–4^. Retinal degeneration, resulting from inherited retinal dystrophies (IRDs), has also been shown to trigger microglial activation^5,6^. IRDs are a heterogeneous group of diseases resulting from genetic changes causing retinal degeneration or dysfunction^7^. These diseases are among the most common cause of blind registrations in developed countries^8^. Recent studies from animal models of IRD have demonstrated that microglia play a key role in photoreceptor degeneration, either directly, by phagocytosing cells, or indirectly, by secreting cytokines which induce photoreceptor apoptosis^4–6,9,10^. Membrane-type frizzled related protein (MFRP) is a transmembrane receptor protein expressed primarily by the retinal pigment epithelial (RPE) and ciliary body in the eye and is expressed in the apical microvilli of RPE. In humans, mutations in the *Mfrp* gene result in an early-onset retinitis pigmentosa associated with microphthalmia, foveoschisis and optic disc drusen in patients. Animal models of *Mfrp* associated retinal degeneration (MARD) have been developed which demonstrate many features of human disease including progressive early-onset retinal degeneration, low electrophysiological responses and microphthalmia^11–13^.

Interestingly, these mice also develop early-onset autofluorescent retinal spots which are thought to be the result of subretinal deposits^14^. Studies have demonstrated subretinal immune cell infiltration showing positive staining for macrophage^15,16^. Recent reports suggest that activation of resident microglia and monocyte derived macrophages could both contribute to subretinal microglia. Resident microglia can migrate to the subretinal region as an adaptive mechanism in response to ongoing damage in retinal degeneration models^4,17^. Some studies have investigated the activation of subretinal microglia in acute-onset retinal degeneration models^18^. On the other hand microglial depletion agent colony-stimulating factor 1 receptor (CSF1R) inhibitor was used to deplete resident microglia, showing that monocyte derived macrophages are recruited to the retina, including the subretina to exacerbate cone death in the rd10 model^19^. However, there has been relatively little exploration of microglial activation and migration in genetic models of human retinal degeneration. In the current study, we used a recently described MARD mouse model carrying homozygous c.498_499insC mutations^13^, to better understand the changes in retinal microglia in these mice. We hypothesize that in this early-onset IRD, resident retinal microglia are activated and are recruited to the subretina. To test our hypothesis, we performed single cell RNA sequencing (scRNA-seq) and validated our findings using immunostaining, coupled with in vivo and ex vivo retinal imaging.

## Results

### Retinal imaging of patients and mice homozygous for c.498_499insC mutations demonstrated discrete white spots and localized to the subretina

**Figure 1:**
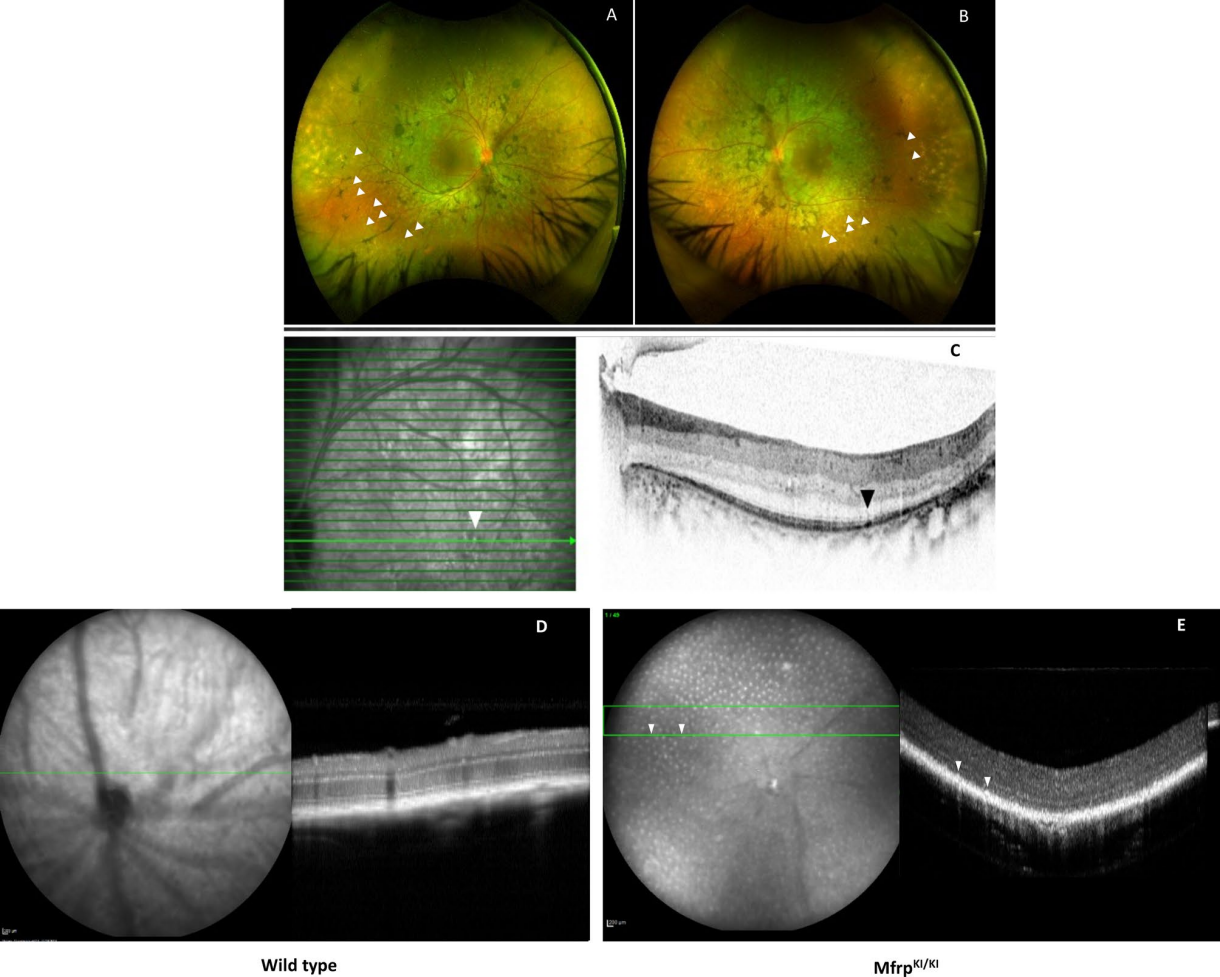
Imaging from a 53-year-old female patient with homozygous c.498_499insC mutations in *MFRP.* (**A,B**) Ultra-widefield pseudo-color imaging from (**A**) right, (**B**) left eyes demonstrate signs of retinal degeneration with symmetrical retinal pigmentation and atrophy with relative sparing of central macula. Additionally, the imaging demonstrates multiple creamy white spots in the far and mid-periphery bilaterally (arrowheads). (**C**) Spectral domain optical coherence tomography aligned with near infrared imaging isolates one of the white spots in the macula (white arrowhead) and demonstrates a subretinal location of this spot (black arrowhead). (**D,E**) SLO images showing multiple autofluorescent spots in the fundus of WT and *Mfrp*^KI/KI^ mice retina.

Four siblings with homozygous c.498_499insC *Mfrp* mutations were previously found to have retinitis pigmentosa, foveoschisis, microphthalmos with hyperopia, and optic disc drusen consistent with MARD. The original imaging studies in this family had primarily focused on central color fundus and electrophysiological changes^13^. However, it was not clear whether the cases also developed a discrete spot phenotype similar to mouse models of MARD. As a result, we performed ultra-wide field color fundus imaging (Fig. 1A–C) which identified discrete creamy-white spots in the mid-periphery and far-periphery of patients (Supplementary Fig. 1). However, subretinal changes were also different from that seen in mice with spots coalesced into a more reticular pattern and not covering the whole retina but located in the far and mid-peripheral retina. Additionally, the spots did not autofluoresce. These studies highlighted a possible subretinal location of deposits in the few cases where occasional spots were found more centrally in the parafovea and peripheral macula and were therefore accessible to SD-OCT imaging.

As this was a novel finding, in order to see that the spots in patients were not mutation specific, and limited to patients harboring the c.498_499insC *Mfrp* mutation, we similarly performed ultra-widefield imaging and SD-OCT in a 44-year-old male patient carrying c.523C > T, p.(Gln175*) and c.649G > A, p.(Gly217Arg) variants in *Mfrp* (Supplementary Table A) who also exhibited classical features of MARD including retinal degeneration, nanophthalmos and foveoschisis. Imaging identified discrete creamy white spots which appeared in a subretinal location. The c.523C > T, p.(Gln175*) mutation has been reported previously and identified as pathogenic. There are 10 individuals heterozygous for this variant in gnomAD. The *Mfrp* c.523C > T, p.(Gln175*) variant generates a premature stop codon in exon 5 (out of a total of 15 exons) and is predicted to lead to loss of normal protein function and is potentially novel. This variant is absent in gnomAD. *MFRP* c.649G > A, p.(Gly217Arg) replaces a moderately conserved amino acid glycine (Gly) to arginine (Arg) at protein position 217 (exon 6). All in silico tools utilized (PolyPhen, SIFT, Mutationtaster) predicted this variant to be damaging to protein structure and function, however as the variant has not been reported before it was classified as a variant of undetermined significance. Family member testing is under way to help further variant classification and phase analysis. Overall, the pattern of the dystrophy is very similar between the new patient and the previously described patients with creamy/white spots mainly seen in the mid to far periphery, suggesting that the subretinal white/creamy spot phenotype is not restricted to a particular mutation in *MFRP* (Supplementary Fig. 2). Fundus imaging in 4–5-month-old *Mfrp*^KI/KI^ mice had shown regular dispersed autofluorescent spots throughout the retina which were co-localized with a subretinal location on SD-OCT. The white spots in humans had a different distribution, as described above, in that there were fewer present in the central macula where fewer rods are known to be found. In the mouse the AF spots were found throughout the retina. The mouse retina is rod dominant with rods distributed throughout the retina with no macula. This suggests that the spots may be associated with a rod distribution^13^ (Fig. 1D,E).

### Immunostaining of retinal cross sections and flow cytometry confirms significant immune infiltration in *Mfrp*^*KI*/KI^ mice retina

**Figure 2:**
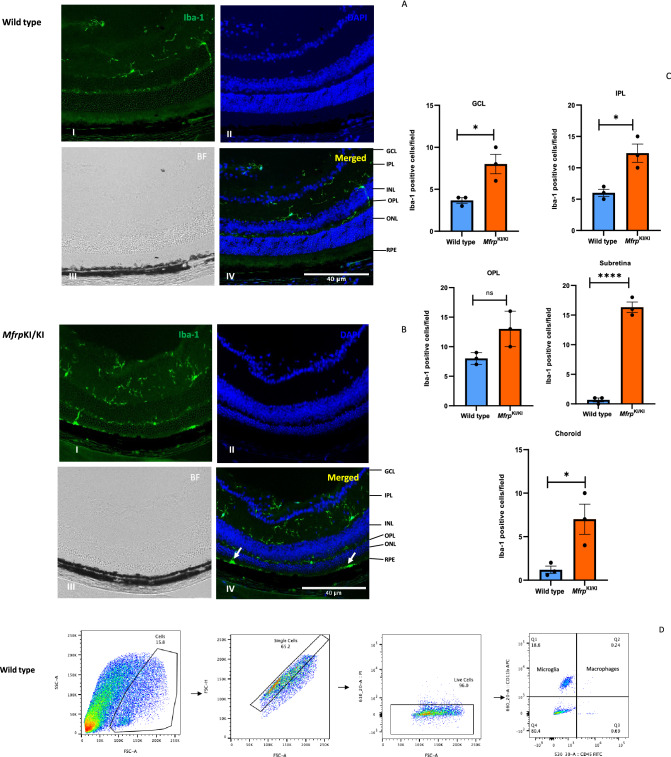

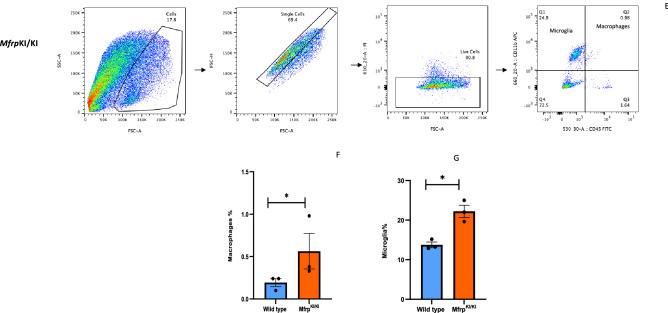
Immunostaining and flow cytometry for microglial and macrophage markers. (**A,B**) Retinal cryosections stained with Iba-1 antibody showed large numbers of positive cells in *Mfrp*^KI/KI^ mice retina (I-Iba-1, II-DAPI, III-Brightfield (BF), IV-Merged) (**B**) particularly in the subretinal region (white arrows) compared with WT mice retinal cryosections (I-Iba-1, II-DAPI, III-Brightfield (BF), IV-Merged) (**A**). (**C**) Significantly higher numbers of microglia were present in *Mfrp*^KI/KI^ mice retina when compared to WT mice retina in the subretina (****p < 0.0001), GCL (*p < 0.05), IPL (*p < 0.05) and choroid (*p < 0.05) region (n = 3). (**D,E**) Flow cytometry of immune cells from mouse retina was performed. First, single cells were gated, followed by live cells. Microglia were gated as CD11b^high^ CD45^low^ cells (Q1) and macrophages as CD11b^high^CD45^high^ (Q2). (F**,G**) There were significantly increased numbers of CD11b^high^ CD45^low^ microglial cells (*p < 0.05) (n = 6) and monocyte-derived CD11b^high^CD45^high^ macrophages in *Mfrp*^KI/KI^ retina compared with WT mouse retina (*p < 0.05) (n = 6) (*GCL* ganglion cell layer, *IPL* inner plexiform layer, *INL* inner nuclear layer, *OPL* outer plexiform layer, *ONL* outer plexiform layer, *RPE* retinal pigment epithelial cell layer).

Retinal cryosections, which were immunostained for Iba-1, demonstrated large cells strongly positive for Iba-1 in the subretina. Iba-1 positive cells were also present in other layers in the *Mfrp*^KI/KI^ mice (Fig. 2A,B). We compared the microglial numbers between WT and *Mfrp*^KI/KI^ and observed significantly greater numbers of microglia in almost all the retinal layers in *Mfrp*^KI/KI^ mice, particularly in the subretina (*p* < 0.0001), GCL (*p* < 0.05), IPL (*p* < 0.05), choroid (*p* < 0.05) (Fig. 2C). Immune cells were also analyzed in retinal cell suspension. Using flow cytometry, microglia were counted as CD11b^high^CD45^low^ and monocyte-derived macrophages as CD11b^high^CD45^high^, as described previously^20–22^ (Fig. 2D,E). We observed a significant increase in microglia (*p* < 0.05) and monocyte-derived macrophages, in *Mfrp*^KI/KI^ mice retina. (*p* < 0.05) (Fig. 2F,G).

### Subretinal microglia accumulate in *Mfrp*^KI/KI^ mouse retina demonstrating strong activation marker expression

**Figure 3:**
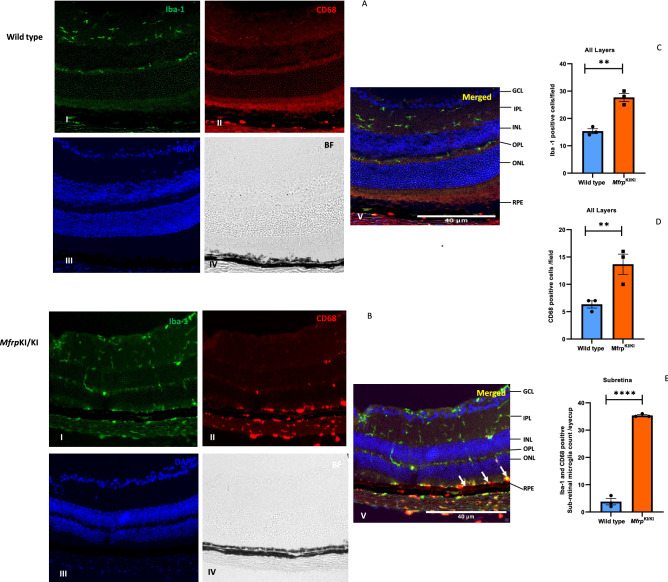
Immunohistochemistry studies for CD68 and Iba-1 positive cells in retinal cryosections (**A,B**) Iba-1(green) and CD68 (red) positive cells were prominent and strongly positive subretinal region (white arrows) and with occasional cells with co-localized immunostaining in the OPL and IPL in *Mfrp*^*KI*/KI^ retina, whereas in WT mice, microglia with Iba-1 cells were confined to the OPL and IPL and completely absent from the subretinal region (I-Iba-1, II-CD68, III-DAPI, IV-Brightfield (BF), V-Merged) and had few cells with CD68 immunostaining. (**C,D**) Iba-1 and CD68 count in both groups (**p < 0.01 and **p < 0.01) (n = 3). (**E**) The number of subretinal microglia were significantly higher in *Mfrp*^*KI*/KI^ retina compared to WT retina (****p < 0.0001) (n = 3) (*GCL* ganglion cell layer, *IPL* inner plexiform layer, *INL* inner nuclear layer, *OPL* outer plexiform layer, *ONL* outer plexiform layer, *RPE* retinal pigment epithelial cell layer).

**Figure 4:**
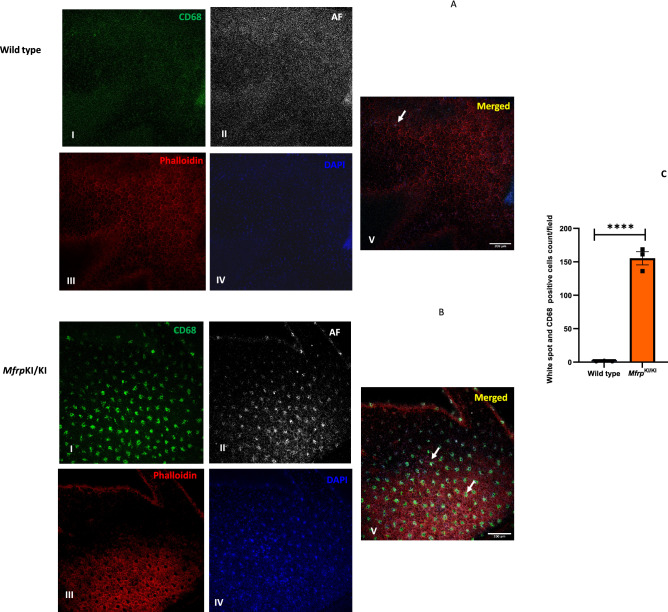
Confirmation of the microglial origin of autofluorescent spots in *Mfrp* ^KI/KI^ mice retina. (**A,B**) Retinal wholemount immunostaining for CD68 positive cells and autofluorescent spots using confocal microscopy with × 20 lens (Right panel), enlarged view of individual spots (I-CD68, II-AF (Autofluorescence), III-phalloidin (for actin, staining) IV-DAPI, V-Merged), (Left panel) show that (**C**) a significantly higher number of autofluorescent spots were present in *Mfrp*^KI/KI^ mice retina (****p < 0.0001) (n = 3).

Microglia in WT retina had a typical ramified appearance, which has been described for quiescent resident microglial phenotypes, in both the retina (Fig. 2A–C) and the brain^23,24^. However, a few ramified microglia were still present in *Mfrp*^KI/KI^ retina, mostly in the inner retina, which was consistent with our scRNA-seq cluster analysis which identified a transcriptionally quiescent resident-type microglia cluster in *Mfrp*^KI/KI^ mice. Immunostaining also demonstrated significantly greater numbers of CD68 and Iba-1 positive cells in *Mfrp*^KI/KI^ mice retina (Iba-1, *p* < 0.01, CD68, *p* < 0.01) compared with WT mouse retina (Fig. 3A,B). There were significantly greater large, round and strongly CD68 positive cells localized to the subretinal region (*p* < 0.0001) in *Mfrp*^KI/KI^ mice retina compared with WT mice (Fig. 3C–E). In WT mice, microglia were mostly present in the IPL and OPL, whereas in *Mfrp*^KI/KI^ microglia were present in the GCL, IPL, OPL, choroid layers and subretina (Figs. 2A, 3A,B). To further investigate the autofluorescent spot phenotype in the *Mfrp*^KI/KI^ mice model, and to confirm that microglia were responsible for this in vivo imaging finding, retinal wholemounts of both WT type and *Mfrp*^KI/KI^ mice were prepared. In the retinal wholemounts we sought to understand whether the autofluorescent spots co-localized with subretinal deposits which were observed during in vivo imaging. Autofluorescent spots on retinal wholemounts were co-localized with CD68 positive activated microglia in *Mfrp*^KI/KI^ mice retina. The findings confirm an immune component to the autofluorescent spots, which were significantly (*p* < 0.0001) greater in number in *Mfrp*^KI/KI^ mice retina when compared with WT type retina (Fig. 4A–C). Taken together, the findings in these set of studies identified activated subretinal microglia which correspond with autofluorescent spots, similar to the ones seen on fundus autofluorescence.

### Microglia in *Mfrp*^*KI*/KI^ mice retina demonstrate increased numbers of cells with an active expression profile

**Figure 5:**
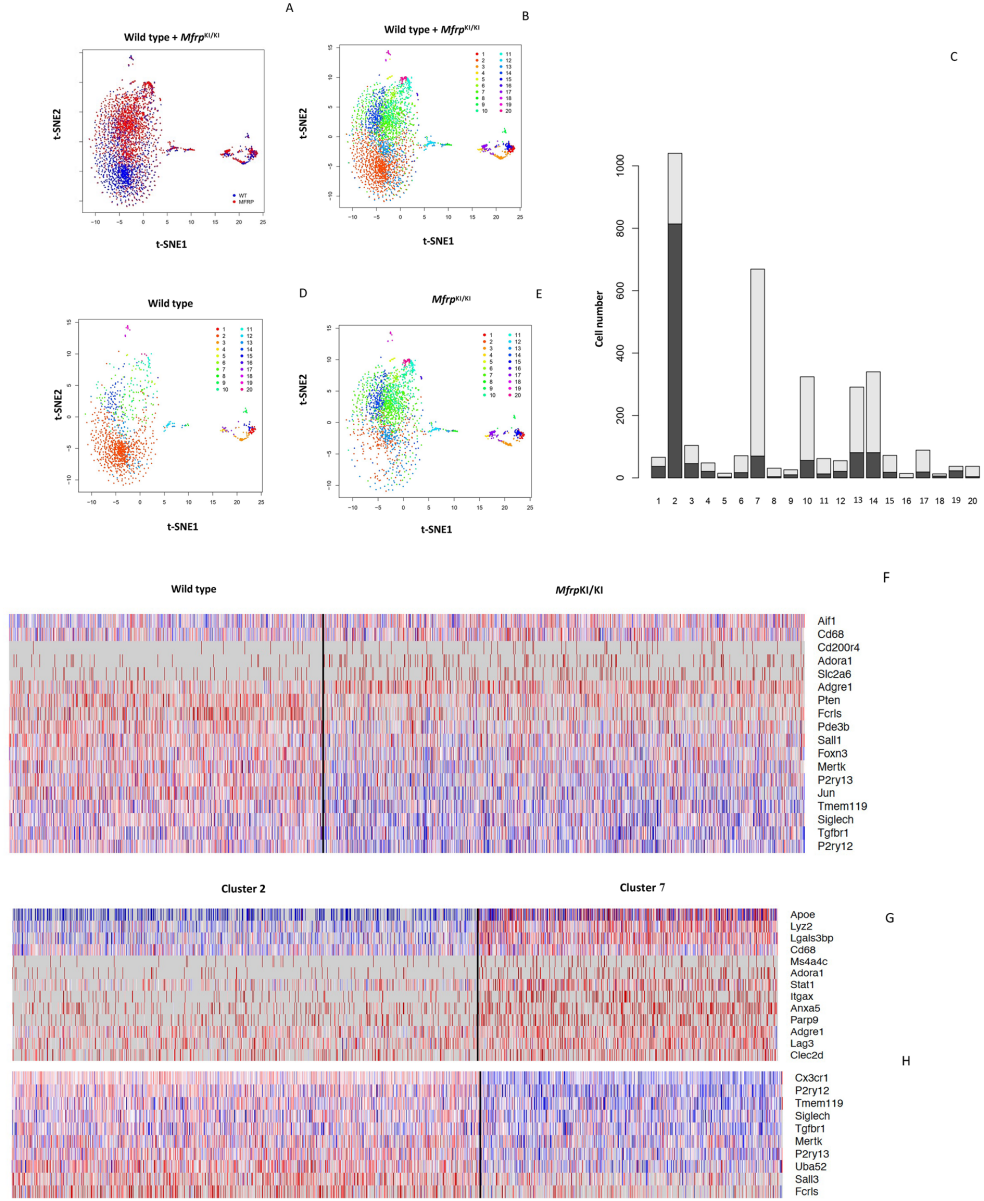

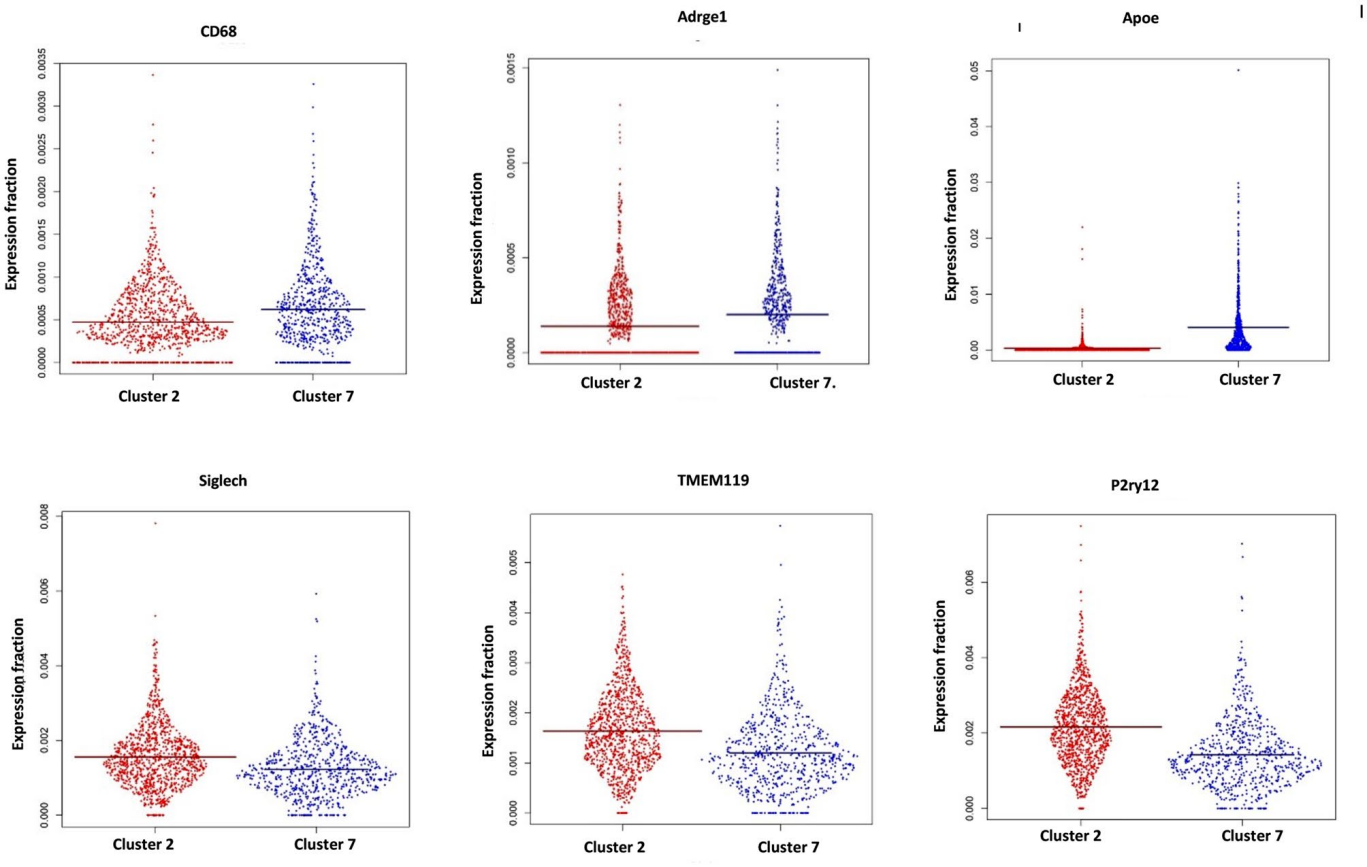
Single cell RNA sequencing analysis. tSNE plots and clustering of microglia from WT (1345 Cells) and *Mfrp*^*KI*/KI^ (2059 cells) retina. (**A**) tSNE plot of microglia from WT and *Mfrp*^*KI*/KI^ mouse retina. (**B**) tSNE plot dividing microglia from WT and *Mfrp*^*KI*/KI^ mouse retina into clusters. (**C**) Histogram representing microglia divided into clusters from both groups, (Black-WT cells, Gray-*Mfrp*^*KI*/KI^. (**D**) WT mouse retina microglia tSNE plot with clusters. (**E**) *Mfrp*^*KI*/KI^ mouse retina microglia tSNE plot with clusters. Heat maps of generated by “pheatmap” package in R program. (**F**) Differentially expressed microglial markers in WT and *Mfrp*^*KI*/KI^ mice. (**G**) Microglial activation, (**H**) homeostatic markers after comparing cluster 2 and 7 (Blue = Downregulation, Red = upregulation). (**I**) Violin plots showing expression of *CD68*, *F4/80*, *ApoE* (microglial activation markers) *Siglech, Tmem119, P2ry12* (homeostatic markers) *lfdr* < 0.1.

In order to better understand the expression profile of retinal microglia in *Mfrp*^KI/KI^ mice, we performed scRNA-seq of CD11b^high^CD45^low^ FACS sorted cells from age-matched *Mfrp*^KI/KI^ and WT type mice retina. Clustering analysis of this data from both *Mfrp*^KI/KI^ and WT type mice retina resulted in the separation of 20 microglial clusters, demonstrating a heterogeneous population numbered from 1 to 20 with cell numbers ranging from 20 to 1000 cells. We first compared *Mfrp*^KI/KI^ and WT type gene expression at a local false discovery rate *(lfdr)* < 0.1 (Fig. 5A–E) and identified significantly increased numbers of microglia expressing microglial activation markers including *CD68* and an increase in other microglial markers such as *F4/80, Iba-1* and decreased numbers of microglia expressing microglial homeostatic markers, associated with quiescent microglia, including *TMEM119*, *Siglech, P2ry13* and *P2ry12* in *Mfrp*^KI/KI^ retina (Fig. 5F) (Supplementary Table B). There was also increased expression of inflammatory markers in *Mfrp*^KI/KI^ as compared to WT (Supplementary Table C). Two clusters contained the highest number of cells; clusters 2 and 7. To identify the phenotype of cells in these clusters we looked at their composition more closely. Cluster 2 was composed of 80.3% WT microglia while cluster 7 was composed of 88.2% *Mfrp*^KI/KI^ microglia. We compared cluster 2 and 7 gene expression and found that cluster 2 had a higher number of cells expressing homeostatic microglial markers including *TMEM119* (*fold change* =  − 1.36, *lfdr* = 1.25E−08), *Siglech* (*fold change* = − 1.27, *lfdr* = 7.97E−05) and *P2ry12* (*fold change* =  − 1.51, *lfdr* = 1.07E−18) and a lower number of cells expressing microglial activation markers including *CD68* (*fold change* = 1.31, *lfdr* = 0.000526) and microglia marker *F4/80* (*fold change* = 1.44, *lfdr* = 0.013281). Hence, cluster 2 appeared to be composed of quiescent retinal microglia, which is likely to explain the greater proportion of cells in cluster 2 from WT mice retina. In contrast, expression characteristics for cluster 7 suggested activated microglia, which is likely to explain why cells in cluster 7 were predominantly derived from *Mfrp*^KI/KI^ mice retina (Fig. 5G–I) (Supplementary Tables D, E).

### *Mfrp*^KI/KI^ retinal microglia had a small cluster of cells with a unique gene signature resembling subretinal phenotypes

**Figure 6:**
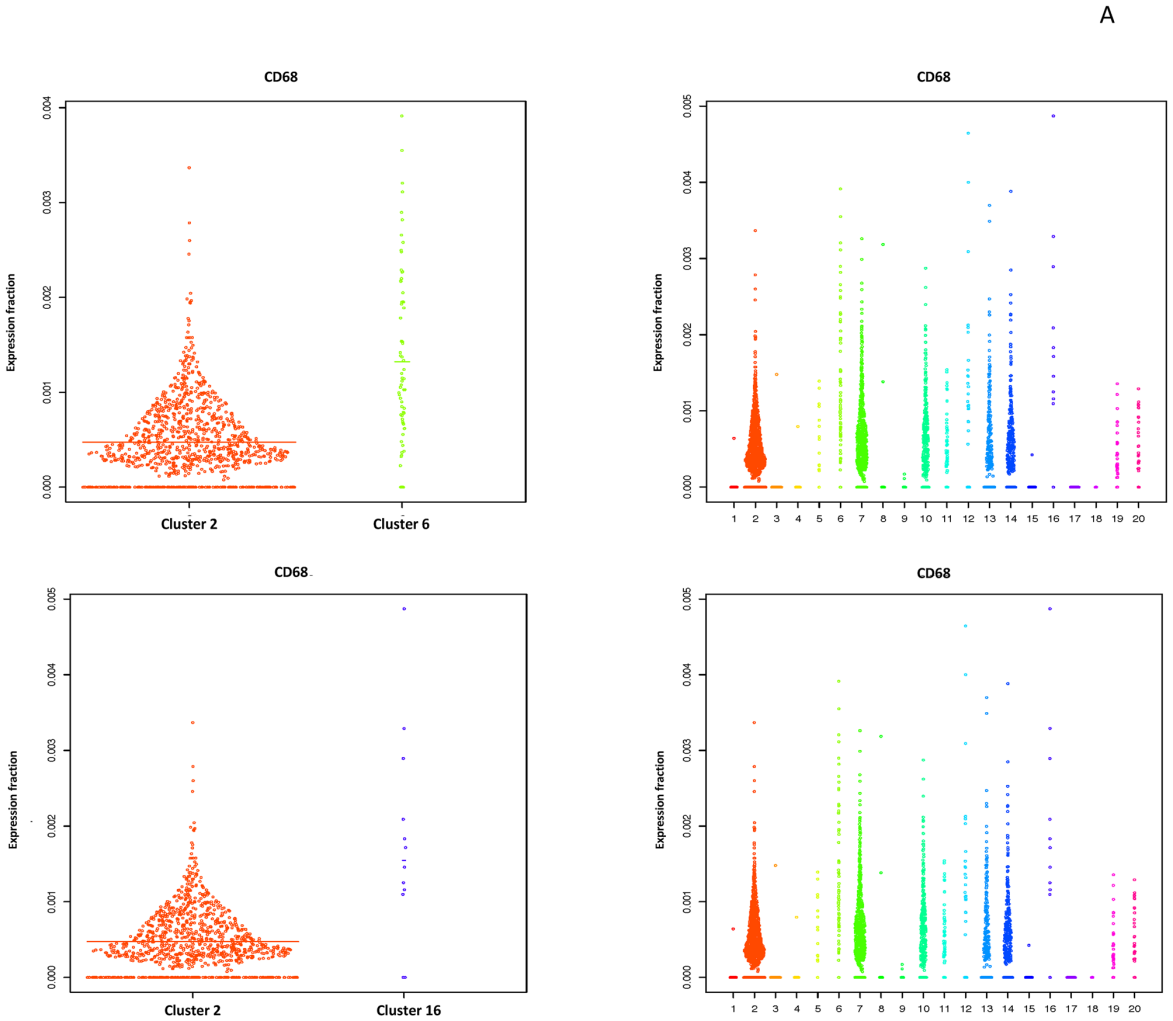

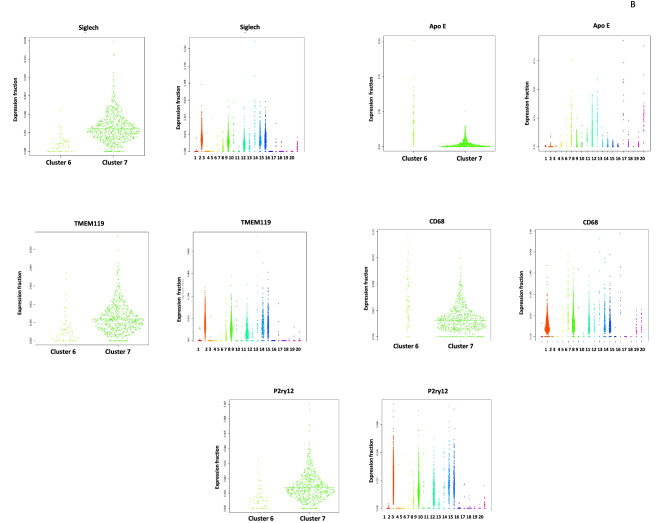
Violin plots: (**A**) comparing CD68 expression cluster 2 with cluster 6, cluster 2 with cluster 16. (**B**) Comparing key genes expressed in clusters 6 and 7.

We next tried to isolate a cluster of cells which represented the activated subretinal macrophages that we identified in our retinal immunohistochemistry studies. Since we found that cluster 7 had greater numbers of activated microglia in the combined *Mfrp*^KI/KI^ and WT analysis (Fig. 5C), we hypothesized that subretinal cells would generate a smaller activated cell population, from our retinal wholemount studies. We first confirmed the characteristics of the two largest clusters, cluster 2 and 7. Cluster 7 showed an active microglial signature present in exclusively in *Mfrp* ^KIKI^ (cluster 7 *CD68* (*fold change* = 1.31, *lfdr* = 0.000526) and cluster 2, mostly inactive microglia present in WT (Fig. 5) (Supplementary Tables D, E). We next compared cluster 2, the quiescent microglial cluster, with all other 19 clusters with a cut off *lfdr* < 0.1. We looked initially for clusters with a high number of cells expressing *CD68* and found that cluster 6 and 16 had high *CD68* expression (cluster 6 (*CD68*, *fold change* = 2.78*, lfdr* = 3.32E−10), and 16 (*CD68*, *fold change* = 3.26, *lfdr* = 0.0015) along with other activation markers and homeostatic static markers downregulation within *lfdr* cut off (Fig. 6A) (Supplementary Tables F, G). Among other clusters which were noted to have a CD68 expression, cluster 5 had *lfdr* value of 0.1 (*CD68*, *fold change* = 1.30 *lfdr* = 0.10), cluster 10 (*CD68*, *fold change* = 1.2 *lfdr* = 0.4) and 12 (*CD68*, *fold change* = 1.5 *lfdr* = 1) had increased *CD68* expression but non-significant *lfdr* values and lastly cluster 11,13,14 had baseline *CD68* expression (*fold change* = 1), clusters 1, 3, 4, 9,15 had low expression of *CD68* with *lfdr* < 0.1 representing inactive clusters and cluster 8, 19, 20 had low expression of *CD68* with a non-significant *lfdr* > 0.1.

As we aimed to identify activated microglial clusters in *Mfrp* ^KI/KI^ mice which may represent subretinal microglia we focused on clusters 6 and 16 and investigated microglial activation and homeostatic markers. We observed that cluster 6 and 16 had cells with increased expression of many myeloid markers. We also analyzed cluster 10, 13 and 14 in detail looking closely with respect to other marker expression (*Iba-1* and *F4/80)* (Supplementary Table H). We found that cluster 10 and 14 were are also active clusters, with a large proportion of cells from *Mfrp* ^KIKI^ retina. We further tried to analyze small candidate in detail and compared cluster 6 with 40 cells and cluster 7 representing largest activated retinal microglia, we compared cluster 7 with cluster 6, one of the active small clusters. We found that cluster 7 had cells having higher expression of resident markers and lowered activation markers compared to cluster 6 *TMEM119* (*fold change* = 1.95, *lfdr* = 0.061), *Siglech* (*fold change* = 2.94, *lfdr* = 8.16E−08) and *P2ry12* (*fold change* = 1.99, *lfdr* = 0.005), *CD68* (*fold change* =  − 2.12 *lfdr* = 1.23E−05) and *ApoE* (*fold change* = − 8.27, *lfdr* = 2.20E−17) (Fig. 6B) Supplementary Table I). These results suggest cluster 6 was one of clusters which could represent subretinal microglia, however other clusters cluster 7, 10, 14 and 16 could also share these active populations and there was a possibility that the subretinal population was not represented by a single cluster but a combination of these clusters representing an active subretinal microglial population with transient states and heterogeneity.

### Subretinal microglia in *Mfrp*^KI/KI^ mice have a well-defined activation trajectory

**Figure 7:**
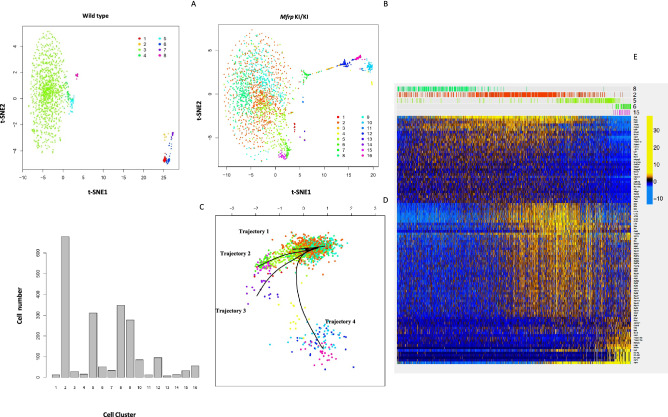
Separate analysis of Microglia from *Mfrp*^*KI*/KI^ and WT mouse retina and trajectory analysis. Microglia from *Mfrp*^*KI*/KI^ and WT mouse retina were clustered separately (**A**) tSNE plot of microglia from WT mouse retina, (**B**) tSNE plot of microglia from *Mfrp*^*KI*/KI^ mouse retina. (**C**) Histogram of clusters generated from *Mfrp*^*KI*/KI^ retinal microglia. (**D**) Trajectory analysis of *Mfrp*^*KI*/KI^ microglia demonstrating four trajectories 1 to 4, Trajectory 1: 8 2 5 4 10 16 12, Trajectory 2: 8 2 9 1 14 13, Trajectory 3: 8 2 5 6 15, Trajectory 4: 8 2 7. (**E**) Heat map, generated by “pheatmap” package in R program, shows gradual changes in gene expression beginning with cluster 8 and ending in cluster 15 in Trajectory 3.

After identifying a candidate cluster of subretinal microglia, we next aimed to better understand activation stages of retinal microglia in *Mfrp*^KI/KI^ mice. We performed clustering of WT and *Mfrp*^KI/K^ retinal microglia populations separately. A total of 8 and 16 clusters were identified in WT and *Mfrp*^KI/KI^ mice respectively demonstrating increased heterogeneity in microglia in the *Mfrp*^KI/KI^ mice (Fig. 7A–C).Microglia from both groups were analyzed in a multidimensional coordinate space defined by relative expression of 18,727 genes describing the state of each cell. We found that *Mfrp*^KI/KI^ microglial cells were less localized, broader and probability density whereas WT microglial cells had a localized probability density (Supplementary Fig. 3A). Further, entropy of distribution function analysis of microglial cells from *Mfrp*^KI/KI^ mice was significantly greater than that from WT mice calculated by creating a random bootstrap ensemble of WT and *Mfrp*^KI/KI^ samples of equal size (Supplementary Fig. 3B).

Transcriptional signature analysis found that retinal microglia in cluster 8 from *Mfrp*^KI/KI^ retina contained mostly resident quiescent microglia like cluster 2 in the original combined WT and *Mfrp*^KI/KI^ analysis. Trajectory analysis was performed, setting cluster 8 as the baseline, to understand potential microglial activation. Analysis resulted in the identification of 4 distinct trajectories labelled as Trajectory 1, Trajectory 2, Trajectory 3 and Trajectory 4. As cluster 8 represented the resident microglia, each trajectory diverged from cluster 8 to the four different branches (Fig. 7D). We analyzed changes in gene expression along each trajectory by serially comparing clusters in each trajectory. Of the four trajectories, only trajectory 3 demonstrated reduced numbers of cells expressing microglial homeostatic markers from cluster 8 and ending at cluster 15, *TMEM119 (fold change* =  − 35.77, *lfdr* = 2.42E−19), *Siglech* (*fold change* =  − 1.74, *lfdr* = 1.35E−07), *P2ry13* (*fold change* =  − 5.65, *lfdr* = 0.0008) and *P2ry12* (*fold change* =  − 10.16, *lfdr* = 4.12E-21*)*. Additionally, the serial analysis showed increasing numbers of cells expressing activation markers, previously found in subretinal cells4, along the trajectory. *Lyz2* (*fold change* = 19.07, *lfdr* = 8.06E−54, *Lgals1*(*fold change* = 1770.32 *lfdr* = 4.24E−07), *CD74* (*fold change* = 167.25 *lfdr* = 9.40E−09) *and F4/80* (*fold change* = 3.13, *lfdr* = 5.98E−07) increased (Fig. 7E, Supplementary Tables J, K). Particularly, there was a marked increase in the number of cells expressing *ApoE* (*fold change* = 38.45 *lfdr* = 2.77E−45). We concluded that cluster 15 in the *Mfrp*^KI/KI^ only analysis and cluster 6 in the combined comparison, each with approximately 40 cells, could represent cells which played a part of the subretinal microglial population, consistent with earlier reports of subretinal cells in RD^25,26^. These results suggest that activated subretinal microglia may result from a stepwise activation of resident retinal microglia in *Mfrp*^KI/KI^ mice retina. These microglial clusters may represent terminally active subretinal microglia with a loss of homeostatic microglial markers but retaining activation marker expression, as has been reported previously^4^.

### Subretinal microglia in *Mfrp*^KI/KI^ retina are activated resident cells strongly express APOE

**Figure 8:**
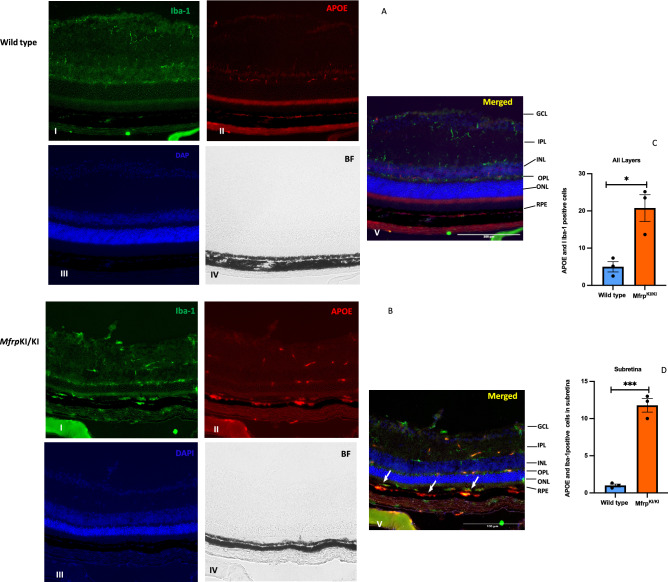
Iba-1 and APOE immunostaining (**A,B**) (I-Iba-1, II-APOE, III-DAPI, IV-Brightfield (BF),V-Merged) was performed, microglial expressing APOE were higher in *Mfrp*^KI/KI^ mice retina compared with WT retina in all layers (**C**) (*p < 0.01) (**D**) in the subretina APOE positive microglia were significantly greater in *Mfrp*^KI/KI^ (***p < 0.001) (n = 3) observed under fluorescence microscope using a × 20 lens and particularly found in the GCL, IPL, INL, OPL and in the subretina (white arrows) (*GCL* ganglion cell layer, *IPL* inner plexiform layer, *INL* inner nuclear layer, *OPL* outer plexiform layer, *ONL* outer plexiform layer, *RPE* retinal pigment epithelial cell layer).

**Figure 9:**
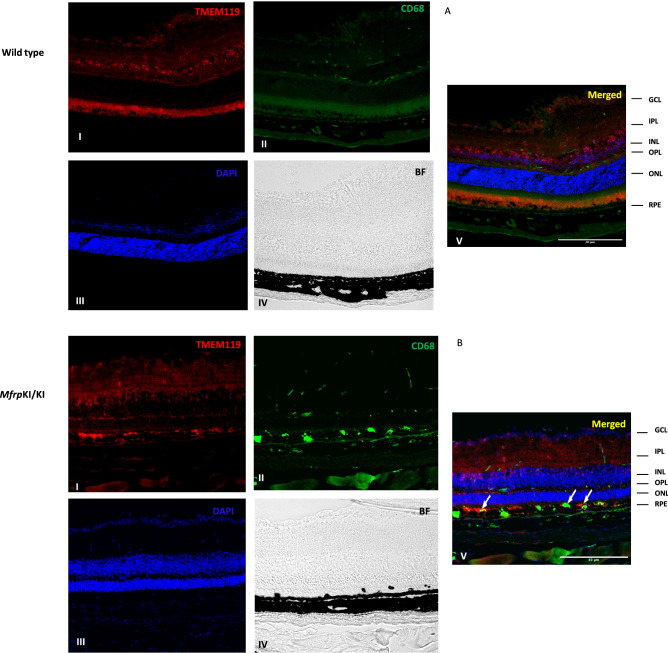
TMEM119 and CD68 immunostaining (A&B) (I-TMEM119-1, II-CD68, III-DAPI, IV-Brightfield (BF), V-Merged) was performed, microglial expressing TMEM119 were higher in WT mice retina compared with *Mfrp*^KI/KI^. In subretinal region TMEM119 and CD68 positive microglia were particularly present in *Mfrp*^KI/KI^ which completely absent from WT (white arrows) (*GCL* ganglion cell layer, *IPL* inner plexiform layer, *INL* inner nuclear layer, *OPL* outer plexiform layer, *ONL* outer plexiform layer, *RPE* retinal pigment epithelial cell layer).

We observed a marked increase in the number of cells expressing microglial activation markers in the scRNA-seq analysis of microglia derived from *Mfrp*^KI/KI^ mice retina, we aimed to validate these transcriptional findings using immunofluorescence in retinal cryosections. Since the trajectory analysis supported a microglial origin of our subretinal candidate cluster, we next performed a validation. The scRNA-seq data had shown that APOE was highly expressed in the microglia, in our candidate subretinal cluster 6. We found significantly higher number of Iba-1/ApoE positive cells in the *Mfrp*^KI/KI^ mice retina compared to WT mice retinal cryosections, when analyzed in all layers (*p* < 0.05), and we also found APOE and Iba-1 co-expressing microglia were present mostly in the subretina (p < 0.001) (Fig. 8A–D) however, not all microglia in the subretina expressed ApoE suggesting heterogeneity in the sub retina. We also found that a significantly greater number of F4/80 positive cells were present in the GCL, IPL, OPL and subretina in *Mfrp*^KI/KI^ retinal cryosections when compared to WT retinal cryosections (*p* < 0.05) (Supplementary Figs. 5, 6) validating our scRNA-seq data. In order to try to confirm that the subretinal cells include cells of microglial origin, we performed immunostaining for resident microglia marker TMEM119 and activation marker CD68. We observed lowered expression of TMEM119 in *Mfrp*^KI/KI^ mice in the GCL, IPL and OPL layers but also found subretinal microglial staining for TMEM119 and CD68 (Fig. 9A,B) suggesting a resident microglial source for some of the subretinal cells.

## Discussion

In the present study, we used a mouse model with homozygous c.498_499insC mutations in *Mfrp.* This pathogenic variant has previously been reported in human cases of MARD^13^. The model shares features of clinical pathology seen in human subjects with homozygous c.498_499InsC. These features include nanophthalmos and rod/cone degeneration. Another key features of *Mfrp* mouse models is the presence of white/creamy spots in retina (Fig. 1A–C)^27,28^. We showed that human cases carrying these mutations also had a distribution of autofluorescent spots in the fundus, using ultra-widefield imaging (Supplementary Fig. 1). Other mutations in *MFRP* have been reported to cause multiple retinal alterations^29^. In the present study, we also described the findings in a 44-year-old male with nanophthalmos associated with high hypermetropia and angle closure glaucoma, who had two different variants in *MFRP,* c.523C > T, p. (Gln175*) and c.649G > A, p. (Gly217Arg), but also demonstrated similar subretinal white/creamy spots suggesting that this finding was not limited to the c.498_499InsC mutation (Supplementary Fig. 2, Supplementary Table A). To our knowledge this is the first report of this white spot phenotype in MARD. This is likely due the relatively recent availability of ultra-widefield imaging.

We also found autofluorescent spots in the fundus of our *Mfrp*^KI/KI^ mice at 4 months of age. The autofluorescent spots had a subretinal localization on SD-OCT imaging (Fig. 1D,E) and were also seen on retinal wholemounts co-localizing with CD68 microglial activation marker (Fig. 4A–C). These findings were consistent with another *Mfrp* mouse model, homozygous for c.174delG mutations^11^ which also have autofluorescent spots in retinal whole mounts, although immune cells in the RPE whole mounts were not co-localized with autofluorescent spots. Another *Mfrp* mouse model, the Rd6 mouse, has been reported to have accumulated phagocytic cells present in the subretina at an early disease onset, as identified by transmission electron microscopy^12^. Similar reports of F4/80 positive microglia contributing to autofluorescent spots have been reported in retinal rosettes of mice with other gene mutations^30^. The *rd7* mouse model carries homozygous R311Q mutations in the *NR2E3* gene and has been shown to have subretinal microglia^31^. It is unclear why the immune cells are found in the subretinal region in the present c.498_499insC *Mfrp*^KI/KI^ model. Immune cells may have been initially recruited to the subretinal region, because of an innate immune response to photoreceptor degeneration. Resident microglia are the major immune cell present in the retina responsible for crucial immune responses to any changes or alterations in the retinal microenvironment. It has been reported that resident microglia or monocyte-derived macrophages can move towards degenerating photoreceptors, the subretina or RPE^32^. Recruitment of microglia to the outer retinal layers, as part of an inflammatory response, has been reported in several retinal pathologies^33^. Complement dependent microglial clearance in the rd10 model leads to neurotoxicity and extensive damage whereas complement deficiency, with lowered microglial phagocytosis resulted in less photoreceptor loss. Thus, microglial activation was thought to be an adaptive mechanism to photoreceptor degeneration^9^. Microglial activation has also been reported to be beneficial, and an adaptive response to protect retina from ongoing damage. One group reported that subretinal microglial protects RPE in a light damage model and ensures proper functioning of photoreceptors^4^. In the *Mfrp*^KI/KI^ mouse model we found a significant increase in overall microglial activation, including subretinal activation, however the contribution of activated microglia to disease progression has yet to be established. To evaluate the role of immune cells, in the current model, we performed immunostaining of retinal cryosections with Iba-1 and observed that there were significantly higher numbers of microglia in *Mfrp*^KI/KI^ mice compared to WT mice retina. Iba-1 positive cells were also present in the subretina (Fig. 2B, panel IV), but were absent in WT mouse retinal cryosections (Fig. 2A, panel IV), suggesting a strong immune response in *Mfrp*^KI/KI^ mice retina. A similar observation was found when microglial numbers were compared in GCL, IPL and the subretina of WT and *Mfrp*^KI/KI^ mice (Fig. 2C). Flow cytometry analysis revealed that microglia numbers were doubled in *Mfrp*^KI/KI^ mice suggesting a strong immune response present shown. There were also increased numbers of monocyte-derived macrophages in *Mfrp*^KI/KI^ retina (Fig. 2D–G). It is not completely clear why there is such a profound cellular inflammatory response in the present model.

We observed that there were more CD68, and Iba-1 positive cells present in GCL, ONL, OPL and IPL region. This typical topological distribution of microglia was not observed in WT retina. Also, WT microglia had a more ramified appearance, which has been reported as a resident quiescent phenotype. Additionally, these quiescent microglia were mainly localized in the IPL and OPL. The number of subretinal microglia were significantly higher in *Mfrp*^KI/KI^ when compared with WT retina (*p* < 0.001) (Fig. 3A–E)^34,35^.

To better understand retinal microglial characteristics and heterogeneity in the *Mfrp*^KI/KI^ mice, we analyzed microglial gene expression by scRNA-seq compared with age-matched WT control. We found *Mfrp*^KI/KI^ microglial cells were highly heterogenous, as analyzed by their distribution in multidimensional coordinate space and entropy distribution (Supplementary Fig. 3A,B). We observed a significant increase in microglial activation markers *CD68* including *F4/80* and *Iba-1* which represent microglia or monocyte-derived macrophages and a decrease in resident homeostatic microglial markers, including *TMEM119, Siglech, P2ry12* and *P2ry13,* in *Mfrp*^KI/KI^ mice retina (*lfdr* > 0.1) (Fig. 5A–I, Supplementary Table B). Retinal degenerative diseases associated with microglial activation have been reported to have a similar gene signature with lowered homeostatic and increased activation markers in the central nervous system and retina^26,36,37^. TMEM119, a transmembrane protein found in microglia, is specifically expressed on the ramified microglial forms and has been used to distinguish homeostatic microglia from monocyte-derived macrophages in the human brain^38,39^ whereas, Siglech, (Sialic acid-binding immunoglobulin type lectins) another well-known marker of resident microglia, exhibits its inhibitory effects by immunoreceptor tyrosine based inhibition motif, downregulating any unnecessary pro-inflammatory immune responses in the retina as well as in brain^40,41^. A change in the local microenvironment can stimulate the loss of these resident microglial markers and increasing expression of CD68 and Iba-1 active forms^42^. Validating our scRNA data, we observed that *TMEM119* expression was lowered in *Mfrp*^KI/KI^ retinal cryosections as compared to WT but that *CD68* and *TMEM119* co-expressing microglia were present in the subretina. This suggests a resident microglial origin for some of the subretinal microglia. These subretinal cells were completely absent from WT retina (Fig. 9A,B).

CD68 has been extensively identified as a microglia activation markers in retinal degeneration studies^43^. Activated microglia express CD68 or macrosialin which is the only known member of the class D scavenger receptors in the retina^44^. We also found a decrease in expression of fractalkine/CX3CR1 in *Mfrp*^KI/KI^ mice, which may explain the increase in microglia and inflammatory marker expression in this model. Another study, using the rd10 mouse model of retinitis pigmentosa, suggested that lowered or altered CX3CR1 expression was associated with increased expression of inflammatory cytokines and microglial activation markers^45^. The role of fractalkine/CX3CR1 signaling has been suggested to play a crucial role in mediating microglia recruitment to apoptotic photoreceptors in the light induced retinal degeneration model^46^. Apart from CX3CR1, other pro-inflammatory cytokines include interleukin (IL)-1 beta, tumor necrosis factor (TNF)-alpha and CCL2, are crucial for microglial induced photoreceptor apoptosis^47^. Previously, Chekuri et al., had reported photoreceptor degeneration because of *Mfrp* mutations, in the *Mfrp*^KI/KI^ model, which may trigger this pro-inflammatory response. To our knowledge microglial induced apoptosis of photoreceptors has not been confirmed in *Mfrp* mouse models, however we observed increased expression of apoptotic markers (*PARP9, ANXA5, TSPO* and phagocytic markers (*CTSB, CTSS, CSTL*) in *Mfrp*^KI/KI^ mice when compared with WT. The presence or absence of apoptosis would be interesting to investigate further in future studies (Supplementary Fig. 4, Supplementary Table L). Next, we aimed to analyze major microglial sub-types in *Mfrp*^KI/KI^ mice retina. We aimed to find gene signatures associated with activated subretinal microglia, described in previous RD models with autofluorescent spots^31^. After confirming that cluster 2 was mostly composed of cells expressing homeostatic microglial markers, and that cluster 7 was composed of relatively more active microglia (Fig. 5G,I) (Supplementary Tables D, E), we looked in more detail at each cluster and found that cluster 6 and 16 (Supplementary Tables F, G), had several upregulated markers previously identified in active subretinal deposits (Lyz2, Ms4a7 and Lgals3bp). Lyz2, is a lysosomal marker, was consistently high in active microglia clusters in all comparisons. It has been reported to be involved in the early stages of microglial activation, in neurodegenerative diseases^26^. Lgals3bp, a cell–cell or cell–matrix interaction modulating protein, along with Lyz2, was high in subretinal active microglia in another study of photoreceptor degeneration model^4^. A recent scRNA-seq study in brain reported a distinct population of cerebral associated macrophages (CAMs) positive for Ms4a7 (membrane-spanning 4-domains subfamily A member 7) present throughout the course of neuroinflammation^25^.

Each of these genes are crucial for processes such as phagocytic clearance of degenerating photoreceptors, as has been reported in the current model. Cluster 6 and 16 could potentially represent subretinal microglia. As cluster 10, 13 and 14 share a good proportion of cells from *Mfrp*^KI/KI^ mice we revisited cluster analysis to find out their characteristics. As previously explained, we first considered CD68 high expressing clusters, but we also tried to investigate other clusters with a high expression of other markers expressed by microglia validated in current study such as Iba-1 or Aif-1, adgre-1 or F4/80 through immunostaining. Coincidentally, we found that cluster 10 had non-significant *CD68.* In addition, cluster 10 also had *Aif-1* or *Iba-1* expression, but had significant adgre1 or F4/80 (*lfdr* < 0.1). Cluster 10 also had cells with a low expression of homeostatic markers (*P2ry13,P2ry12* and *TMEM119).* Other markers known to be high in the activation state (*Lgals3bp, Ms4a7, Ms4a6c),* were highly expressed (*lfdr* < 0.1). This suggests that cluster 10 could be made of active microglia with a different expression profile present in *Mfrp*^KI/KI^ mice compared to WT as we also found adgre-1 or F4/80 positive cells present in the subretina of *Mfrp*^KI/KI^ mice (Supplementary Figs. 5, 6). Cluster 13 also had a relatively good population of cells, but with a low or insignificant expression of activation markers *Iba-1, Aif-1*and *adgre-1* or *F4/80* which suggests these could be microglia in an intermediate state. Similarly, cluster 14 had a higher expression of Aif-1/iba-1, with a large number of cells expressing activation markers (*Lyz2* and *Lgals3bp*) suggesting that a further subset of active microglia exist in *Mfrp*^KI/KI^ mice retina (Supplementary Table H). In summary, we conclude that microglia in *Mfrp*^KI/KI^ mice are present in several activation states and our studies suggest increased heterogeneity in the *Mfrp*^KI/KI^ mice retina as discussed earlier (Supplementary Fig. 3A,B).

We next compared cluster 7, which we define as most active large size cluster, with cluster 6 which was one of small candidate active clusters. We observed that, apart from a significantly higher number of cells expressing known activation markers, there were also other cells expressing myeloid markers (*Lyz2, Ms4a7, Lgals3bp*) (Fig. 6B, Supplementary Table I). We next performed a trajectory analysis to understand activation stages of microglia in *Mfrp*^KI/KI^ mice retina (Fig. 7D). Trajectory 3 had shown increased activation marker expression beginning from putative resident microglial cluster 8 then 2, 5 and 6, finally ending in cluster 15. The progression along the trajectory resulted in a stepwise loss of homeostatic markers, along with higher expression of subretinal markers *Lyz2, Ms4a7, Lgals1* and *ApoE* (Fig. 7E, Supplementary Tables J, K). Microglial activation results in their migration into an injury site, where they phagocytize injured photoreceptors. These findings provide an insight into the microglial response and function during RD in the *Mfrp*^KI/KI^ model. We hypothesize that activated subretinal microglia remove potentially damaging cell debris^15,48^. We also observed that there was a significant increase in cells expressing genes associated with phagocytosis and apoptosis pathways in *Mfrp*^KI/KI^ retinal microglia (PARP9, ANXA5, TSPO) (Supplementary Fig. 4, Supplementary Table L). Poly-ADP ribose polymerase (PARP) is known to be activated as a result of DNA damage, in another model retinal degeneration model, the rd10 model^49^. High cGMP levels in dying photoreceptors were found to correlate with increased activity of PARP^50^. We also observed high *PARP14* and *PARP9* in *Mfrp*^KI/KI^ microglia along with *ANXA5*, which contributes to αvβ5 integrin-dependent photoreceptor phagocytosis clearance at the apical phagocytic surface of the RPE^51^. Similarly, translocator protein (TSPO), was also high in the *Mfrp* ^KI/KI^ microglia, which promotes ROS production, by increasing calcium levels and hence NADPH oxidase 1 (NOX1) activation. TSPO knock out has resulted in retinal protection from angiogenesis and inflammation^35^.

We validated scRNA-seq findings using immunofluorescence of retinal cryosections. We observed that greater numbers of F4/80 positive cells were distributed in the GCL, IPL and OPL, in *Mfrp*^KI/KI^ (Supplementary Fig. 5). We observed prominent F4/80 positive cells present in *Mfrp*^KI/KI^ subretina, whereas no cells were detected in WT retina (Supplementary Fig. 6). In addition, we had noted the higher number of cells expressing APOE, which plays important role in lipid metabolism and it has been reported in subretinal microglia in other retinal degeneration models^37^. Co-staining of Iba-1/APOE in retinal cryosections was performed (Fig. 8A,B). We observed Iba-1/APOE positive cells, which were prominently present in *Mfrp*^KI/KI^ subretina, which were completely absent from WT mice, which appears to validate findings of some of our candidate clusters in our scRNA seq data (Fig. 8C,D). Not all of the subretinal cells were positive for APOE, suggesting a heterogenous population. This warrants further study, and investigation as to the potential source of these other cells. Of note, other cells in the retina, including glial cells such as astrocytes also express APOE^52^. APOE positive cells were also found in the subretina, IPL and OPL in *Mfrp*^KI/KI^ mice retina. We observed a higher number of microglia expressing *APOE* in *Mfrp* ^KI/KI^ retina in scRNA-seq. APOE is a cholesterol transporter associated with retinal degeneration and hence its accumulation in the subretinal microglia has been reported to exacerbate RD^53,54^.

Although our study compared findings in a mouse model of MARD with those of age-matched mice on the same background, the present study only looked at the mice at one age point. This age-point was chosen as this was the approximate age at which degeneration was shown to be at a maximum, from previous longitudinal studies. However, retinal degeneration is not a single event and may change with the stage of degeneration. It would be important to look at earlier age points to confirm our trajectory analysis and to identify triggers for microglial activation. In addition, these studies show an association of immune cells with RD. They do not confirm causation, although expression markers for phagocytosis do suggest that they are involved in photoreceptor phagocytosis. The present study also identified an increased number of monocyte-derived macrophages in the *Mfrp*^KI/KI^ retina using flow cytometry. Further, study will be required to understand the role of monocyte-derived macrophages in MARD. The presence of a heterogenous population of microglia likely represents a changing microenvironment in the retina leading to dynamic changes in the immune response with ongoing RD. In future studies, validation of these markers will help better understanding of the immune response. Taken together, the validation studies confirm the findings from flow cytometry and scRNA-seq data demonstrating an increased number of activated microglia in *Mfrp*^KI/KI^ mice retina compared to age-matched WT mouse retina. In addition, the immunostaining also appears to confirm the scRNA-seq findings that cells in cluster 6 are likely to make up part of the subretinal microglial population seen in the *Mfrp*^KI/KI^ mice, as the subretinal microglia in *Mfrp*^KI/KI^ mice demonstrate strong CD68, F4/80 and APOE expression. However, the subretinal microglial population is heterogeneous. Further microglial knockout, targeted depletion or suppression studies will likely be required to confirm the role of retinal microglia in degeneration in *Mfrp*^KI/KI^ mice.

## Materials and methods

### Experimental animals

All animal procedures were performed with approval by the University of California, San Diego, Institutional Animal Care and Use Committee (IACUC). All methods were conducted in accordance with relevant guidelines and regulations, including the National Institute of Health (NIH), the Association for Research in Vision and Ophthalmology (ARVO) and Animal Research: Reporting of In Vivo Experiments (ARRIVE). *Mfrp*^KI/KI^ mice (4–5 months old) were generated on C57BL/6J background and have been previously characterized^13^. In summary, the c.498_499insC mutation in exon 5 of *Mfrp* gene was generated by targeting exons 3–9, using site directed mutagenesis. The targeting vector (15.25 kb) was generated by sub-cloning a 11.15 kb region from a C57BL/6 BAC clone (RP23:270P20) having exons 1–13 exons *Mfrp* with a LoxP site upstream (exon 3) and a LoxP/FRT‐flanked Neo cassette downstream (exon 9). Age matched WT mice were used as controls. All mice were housed in standard condition at standard temperature (25 °C) and with a 12-h light: dark cycle on a standard diet. All mice were genotyped for the above-mentioned mutation using tail tip samples for DNA before study.

### Clinical evaluation of human patients with *Mfrp* mutation

Institutional Review Board (IRB) approval was acquired from the University of California, San Diego for the review of patient’s data. Informed consent from patients or their legal gradians were obtained as per institutional protocol for participation and publication of information including images. All data and images were anonymized for patient’s safety. All patient studies were conducted in accordance with the protocol approved by the University of California, San Diego and as per the Declaration of Helsinki (https://www.wma.net/policies-post/wma-declaration-of-helsinki-ethical-principles-for-medical-research-involving-human-subjects/). Human subjects with c.498_499insC mutations were examined and had fundus imaging as described earlier^13^. Four siblings of a consanguineous Mexican family with confirmed homozygous c.498_499insC mutations in *MFRP* were recruited at the Institute of Ophthalmology, “Conde de Valenciana” Faculty of Medicine, UNAM, Mexico City for the study. Spectral domain-optical coherence tomography (SD-OCT) imaging of the fundus was performed using SD-OCT Spectralis^®^ HRA + OCT (Heidelberg Engineering, Heidelberg, Germany) at 850 nm wavelength in all patients. Patients also underwent, ultra-widefield pseudo color and autofluorescence imaging (Optos, Dunfermline, United Kingdom). A further 44-year-old male patient was examined at the Shiley Eye Institute, University of California using the same imaging techniques and was confirmed to have the carrying c.523C > T, p.(Gln175*) and c.649G > A, p.(Gly217Arg) mutations in *MFRP* using whole exome sequencing.

### Autofluorescence and optical coherence tomography (OCT) imaging of *Mfrp*^KI/KI^ mice

Fundus imaging of *Mfrp*^KI/KI^ and WT (4 months old, n = 3) mice was performed as described earlier^13^. In brief, mice were anesthetized with intraperitoneal injection of ketamine (93 mg/kg) and xylazine (8 mg/kg), followed by1% atropine and 0.5% tropicamide application to eyes for dilating the pupil. Autofluorescence and OCT imaging of both the eyes were performed at 488 nm excitation SLO and 850 nm wavelength using Spectralis™ HRA + OCT ((Heidelberg Engineering, Heidelberg, Germany) and analyzed by using Heidelberg eye explorer software V2.

### Fluorescence activated cell sorting (FACS)

Microglia were sorted from the retina of each group (n = 6) separately as described with some modifications^55,56^. In brief, mice were euthanized by carbon dioxide inhalation and death was confirmed by cervical dislocation. Eyecups were collected in ice cold 1xphosphate buffer saline (PBS). Eyes were dissected under microscope removing lens and cornea, and retinae were dissociated mechanically in 5 ml cold PBS. The resulting cell suspension was passed through wide bore pipette several times before passing through 70 µm cell strainers to remove large cell clumps. Cells were pellet down at 400 rcf at 4 °C for 5 min and resuspended in 1 ml FACS buffer containing 3% fetal bovine serum in 1× PBS. 1 × 10^6^ cells from each group were stained with rat anti-mouse CD16/CD32 (Mouse BD Fc Block™, BD Pharmingen™, 553141, 1:500) for 20 min at 4 °C followed by staining with anti-CD45 (Biolegend 103108, 0.25 µg per/ul), anti-CD11b (Biolegend, 101212, 0.25 µg per/µl) for 30 min at 4 °C in the dark. Cells were washed in FACS buffer and resuspended in 500 ul 1× PBS containing 0.1% Bovine serum albumin (BSA). Propidium iodide was used to exclude the dead cells during gating, and compensation beads (UltraComp eBeads, Invitrogen, 01-2222) were used to set the experimental compensation per manufacturer instructions. Microglia were sorted as a CD11b^high^CD45^low^ population in 500 µl 1× PBS containing 40 U/ml RNAase (New England Biolabs, M0314S) and 0.04% BSA in BDFACS Aria II using BDFACS Diva™ softwareV9. Experiments were repeated three times (n = 3) and analyzed using FlowJo™ softwareV10.8 to. Doublets were excluded first, and single live cells were gated followed by CD11b^high^ CD45^high^ as monocyte-derived macrophages and CD11b^high^ CD45^low^ cells as microglia population. Percentage of microglia and macrophages were calculated out of live cells taken as parent population in each group.

### Single cell RNA sequencing

FACS sorted retinal microglia (7000 cells) from *Mfrp*^KI/KI^ and WT mice (4-month-old) were processed for ScRNA-seq using 10× Genomics chromium controller and chromium single cell 3ʹ v3 reagent kit as per manufacturer’s instructions (10× Genomics) and libraries were sequenced using an Illumina NovaSeq 6000 sequencer. Initial data was analyzed by Cell Ranger Single-Cell Software Suite (10× Genomics). A total of 1345 cells from WT and 2059 cells from *Mfrp* ^KI/KI^ mice had passed the quality control criteria based on Cell Ranger software. There were three final output files each for gene count, barcodes, and feathers. A pre-built mouse transcriptome reference file refdata-cellranger-mm10-3.0.0 provided by 10×  (https://support.10xgenomics.com/single-cell-gene-xpression/software/downloads/latest) was used for mapping of reads to transcripts. Pairwise distances between cells were calculated as the Jensen-Shannon distances based on per cell gene count frequencies as in Ref.^57^. 20 communities (clusters) were found among the cells using the full distance matrix using affinity propagation algorithm. Communities were visualized using t-SNE dimensional reduction^58^. Heterogeneity in each group was analyzed by calculating probability density in multi-dimensional coordinate space and entropy measurement by creating a random bootstrap ensemble of WT and Mfrp *Mfrp*^KI/KI^ samples of equal size 1345 cells. Differential expression between clusters was assessed with the bootstrap method of Pollard and van der Laan^59^, with *lfdr* calculated using the empirical Bayes method of Efron^60^. A cutoff of *lfdr* = 0.1 was chosen to define significant differential gene expression in *Mfrp* ^KI/KI^ and WT mice. Additionally, cells from *Mfrp*^KI/KI^ and WT mice each were also clustered separately to perform trajectory analysis using the *Slingshot* method^61^. Heat maps were generated by “pheatmap” package in R program (https://www.R-project.org) showing differential expression of genes, red color for upregulated and blue color for downregulated genes respectively.

### Immunofluorescence

Eyes were removed after mice were euthanized by carbon dioxide inhalation and death confirmed by cervical dislocation. Eyecups were fixed immediately in 4% paraformaldehyde for 24 h at 4 °C. After washing in PBS, Eye cups were cryoprotected in sucrose gradient (from 10, 20 to 30%) for 2 h each followed by embedding in Optimal cutting temperature (OCT) compound and cut into 12 µm thin cryosections. For immunodetection of Iba-1, F4/80and co-staining of Iba-1and CD68 sections were fixed in cold acetone for 10 min and washed with 1xPBS followed by blocking in 5% normal goat or donkey serum prepared in PBST (0.1% triton in 1× PBS) for 45 min at room temperature (RT). Further, sections were incubated with primary antibodies: CD68 (rat-anti mouse, 1:500;MCA1957), Iba-1 (rabbit anti-mouse, 1:1000; ab153696), F4/80 rat anti-mouse (1:200; AB664) overnight at 4 °C. Further, for APOE (rabbit anti-mouse, 1:100, ab183597) and Iba-1 (goat anti-mouse, 1:500, 011-27991,Wako chemicals) co-staining and for TMEM119 rabbit anti-mouse, 1:500 ab209064), and CD68 (rat-anti mouse, 1:500;MCA1957) co-staining slightly modified protocol was used. In brief, cryosections were first washed with 1× PBS then incubated for 1 h with 1% sodium borohydride in PBS for antigen retrieval Blocking was done in 5% BSA and 5% donkey serum in PBST (0.3% triton in 1 × PBS) for 1 h at RT. Further, sections were incubated with primary antibodies: Iba-1 (goat anti-mouse, 1:200; AB5076), and APO-E (rabbit anti mouse, 1:200; ab183597) at 4 °C overnight. Antibodies were diluted in 1% BSA in PBST v.

In a separate experiment another F4/80 primary antibody (rabbit anti- mouse 1:500, 70076 Cell signaling) was used for detection with different antigen retrieval system. In brief, sections were incubated with for 1 h (Iba-1/ApoE) or 10 min (TMEM119/CD68) in 1% sodium borohydride in PBS at RT for antigen retrieval and blocked with 3% donkey serum, 0.01% azide and 0.3 M Glycine for 1 h followed by incubation with primary antibody overnight at 4 °C. Compatible secondary antibodies (1:750) were used for detection at RT for 2 h diluted in 1% BSA in PBST. Sections were mounted in vectashield mounting medium with DAPI and all images were acquired under × 20 magnification using Keyence Z-BX800 microscope (Keyence, Itasca, IL). For analyzing single immunodetection three retinal cryosections from each mouse (n = 3) in each group were counted in individual retinal layers. For CD68/Iba-1 and all APOE/Iba-1 positive cells in the subretina we counted 3 complete eye cups each mouse (n = 3) from both group by a person blinded to study conditions.

### Retinal wholemount immunostaining

Retinal wholemount staining was performed as described earlier^62^. Briefly, both the eyes were collected after mice were euthanized by carbon dioxide inhalation and death was confirmed by cervical dislocation. Eyecups were immediately fixed for an hour in 4% PFA at 4 °C, followed by fixation in 70% alcohol for another hour. Eyes were then dissected in cold 1× PBS under dissection microscopes, cornea, lens and vitreous were removed carefully. Retinae were blocked with 3% milk and 3% BSA in 1× PBS for an hour. Further, incubated in rabbit anti mouse CD68 antibody (rat-anti mouse, 1:500; MCA1957) in PBS 1%BSA solution overnight at RT. Retina were rinsed three times in 1xPBS (20 min each) and incubated with secondary antibody (Alexa fluor 488,1:500; A32790) and Rhodamine-Phalloidin (Thermo scientific, R415:1:10) for an hour at RT. After three final washings with PBS (20 min each), each retina was flattened using four radial incisions under light microscope and mounted with vectashield antifade mounting medium with DAPI. Autofluorescent spots were detected at 670 nm excitation and 450 nm emission range. Autofluorescent and Iba-1 positive cells colocalization images were acquired under × 20 magnification using a Nikon AIR confocal microscope by Z-stacking and the closest images to the RPE was used for analysis. Three retinal wholemount images from each group were analyzed for quantitative comparison (n = 3).

### Statistical analysis

All data were compared using unpaired student t-test and presented as mean ± SEM. Data was analyzed using Graph Pad Prism 9.0 (La Jolla, CA, USA) and statistical significance was considered at *p* < 0.05.

### Ethical declarations

All methods and experimental procedures involving animal were performed in accordance with the protocols approved by the University of California, San Diego, USA, Institutional Animal Care and Use committee (IACUC), relevant guidelines and regulations including the National Institute of Health (NIH), the Association for Research in Vision and Ophthalmology (ARVO) and Animal Research: Reporting of In Vivo Experiments (ARRIVE). All patient studies were conducted in accordance with the protocol approved by the University of California, San Diego and as per the Declaration of Helsinki (https://www.wma.net/policies-post/wma-declaration-of-helsinki-ethical-principles-for-medical-research-involving-human-subjects/).

## Supplementary Information

Supplementary Information.
