# Supplementary material for: Single cell RNA sequencing confirms retinal microglia activation associated with early onset retinal degeneration
Source: Sci Rep. 2022 Sep 10;12:15273. doi: 10.1038/s41598-022-19351-w (PMC9464204; doi:10.1038/s41598-022-19351-w)
Supplement: Supplementary file 1 — Supplementary Information. [file 41598_2022_19351_MOESM1_ESM.pdf]

Supplementary Figures

Fig 1

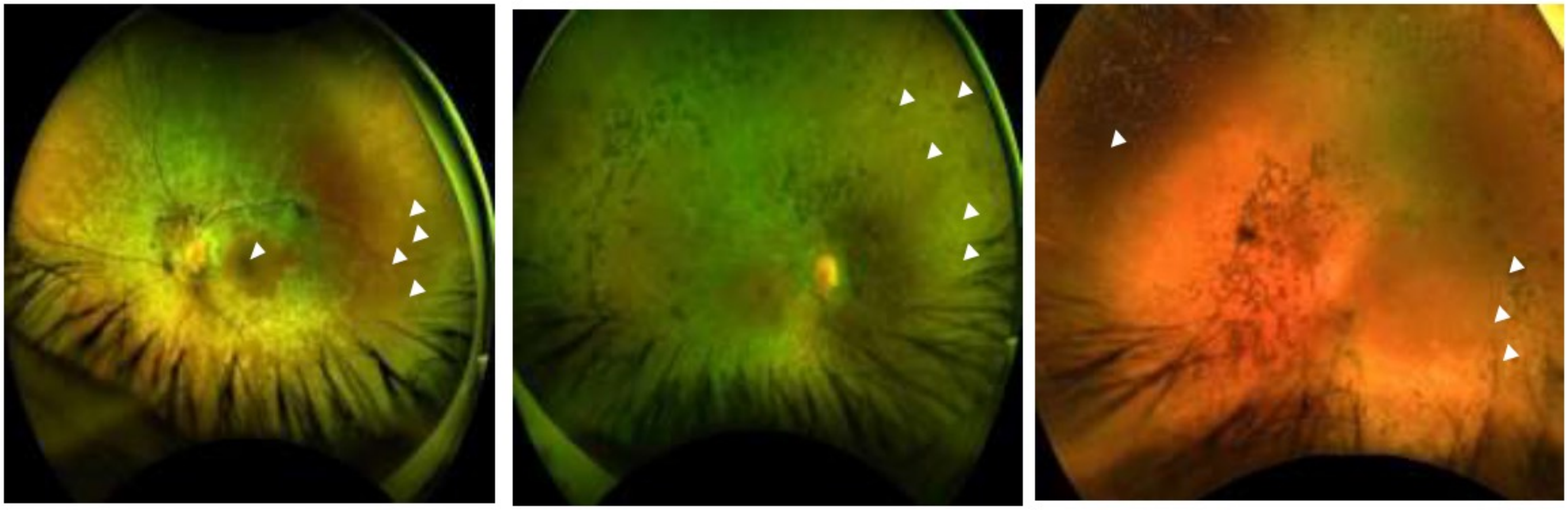

Fig 2

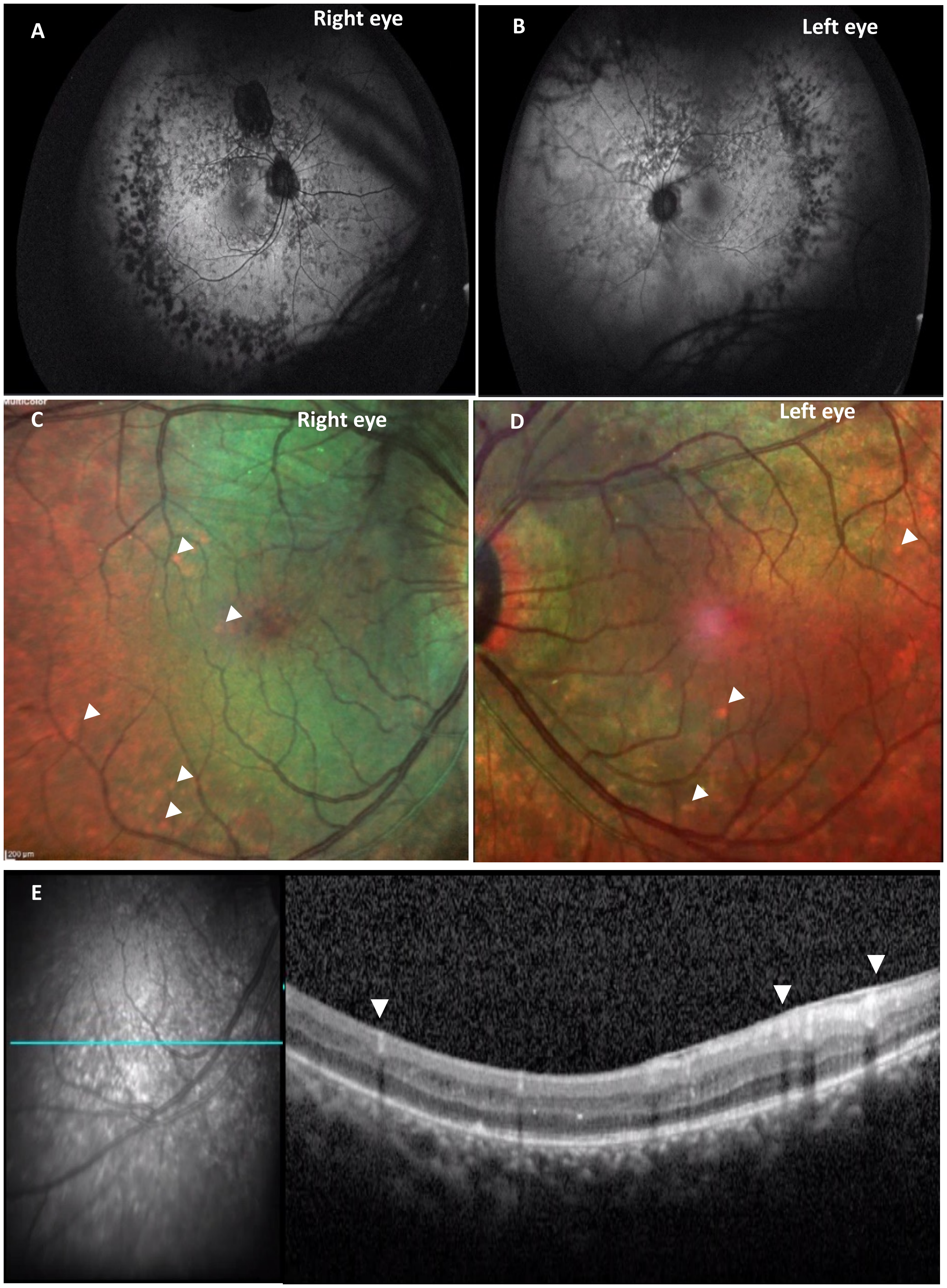

Fig 3

A

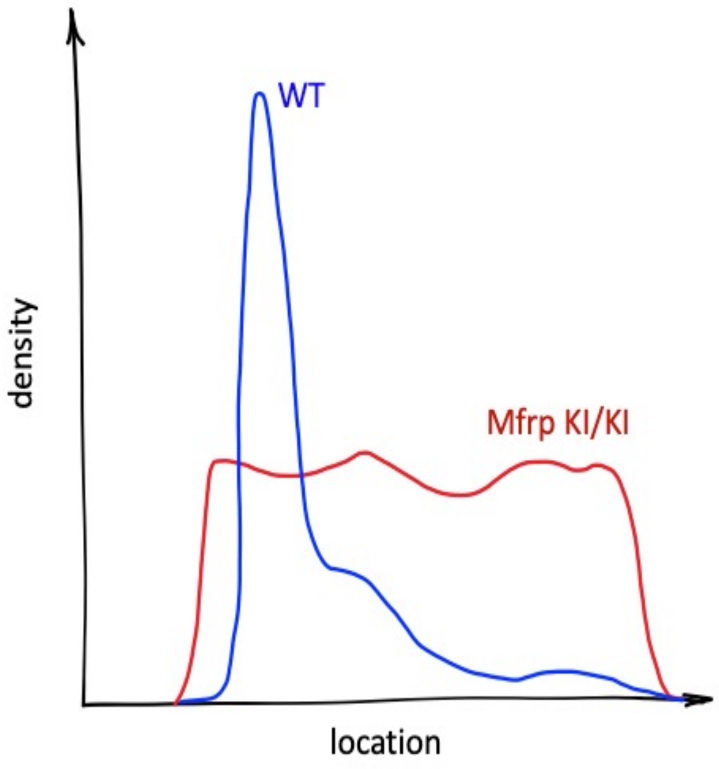
WT
Mfrp KI/KI
density
location

B

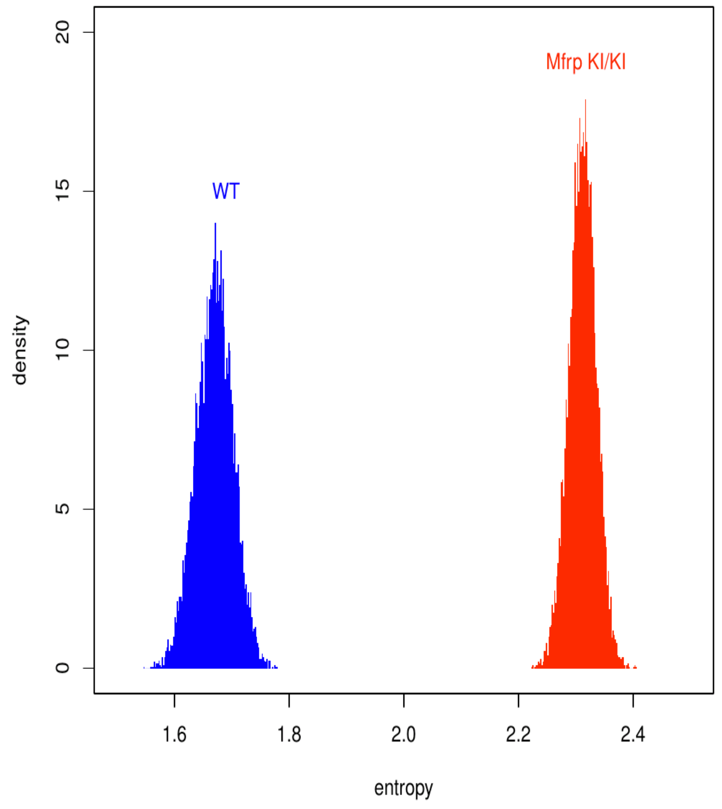
WT
Mfrp KI/KI
density
0
5
10
15
20
1.6
1.8
2.0
2.2
2.4
entropy

Fig 4

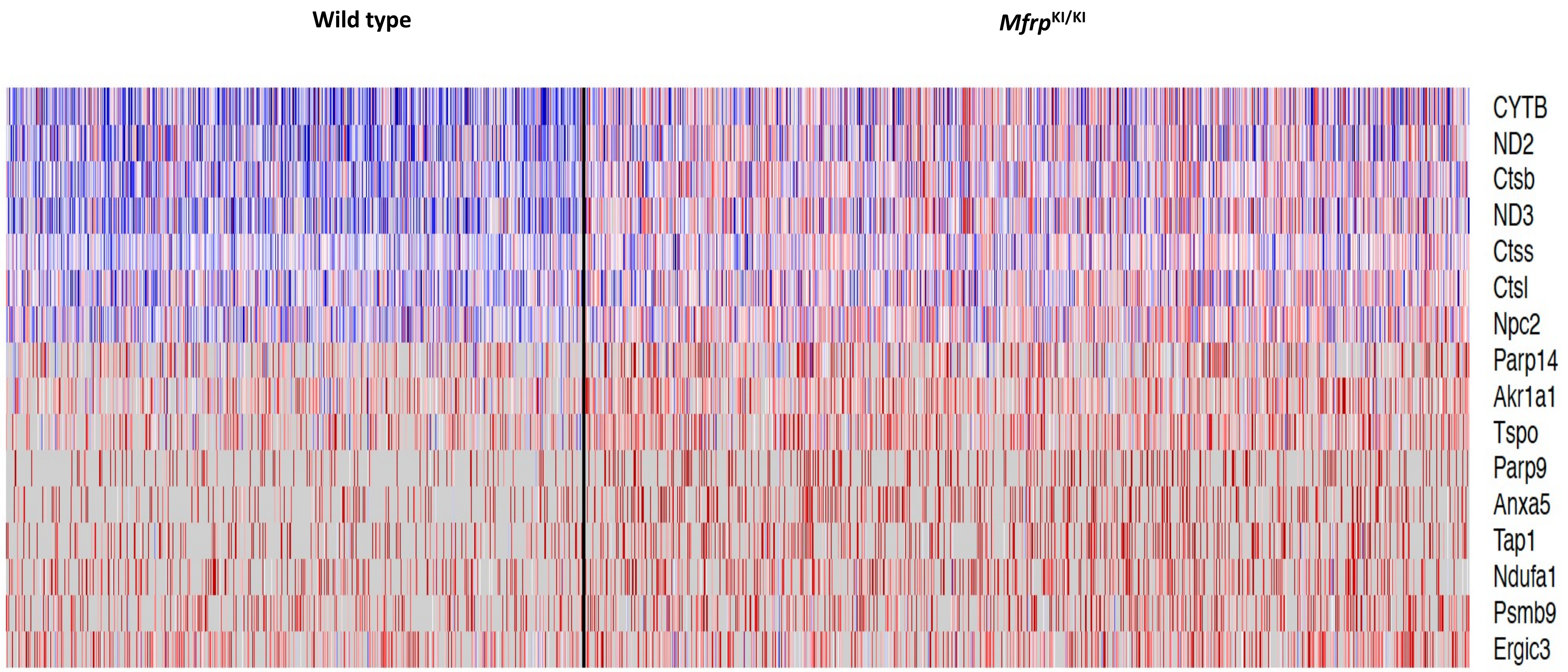

Wild type
Mfrp KI/KI
CYTB
ND2
Ctsb
ND3
Ctss
Ctsl
Npc2
Parp14
Akr1a1
Tspo
Parp9
Anxa5
Tap1
Ndufa1
Psmb9
Ergic3

Fig 5

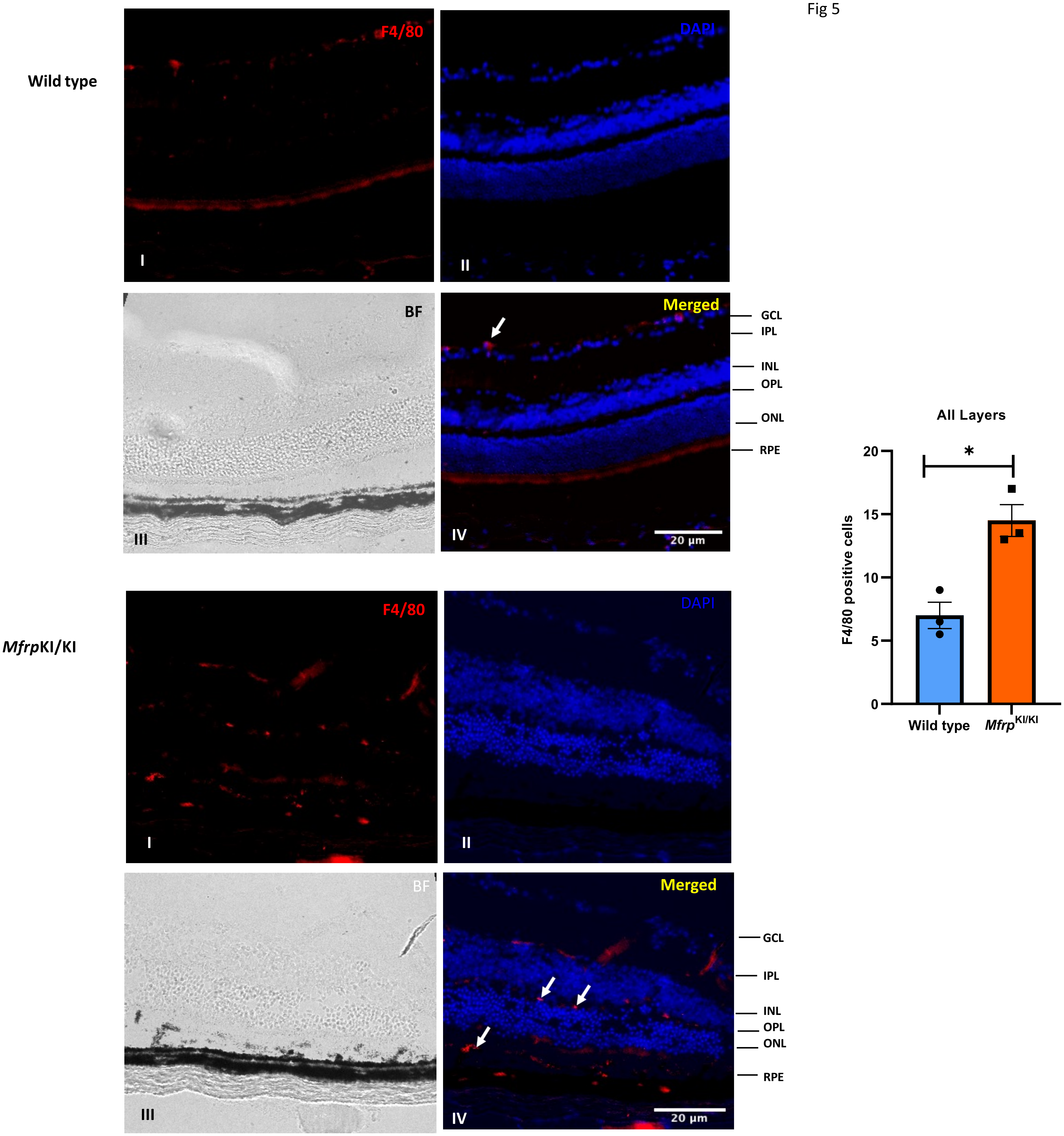

Wild type
F4/80
DAPI
I
II
BF
Merged
III
IV
20 μm
GCL
IPL
INL
OPL
ONL
RPE
*Mfrp*KI/KI
F4/80
DAPI
I
II
BF
Merged
III
IV
20 μm
GCL
IPL
INL
OPL
ONL
RPE
All Layers
F4/80 positive cells
20
15
10
5
0
*
Wild type
*Mfrp*KI/KI

Fig 6

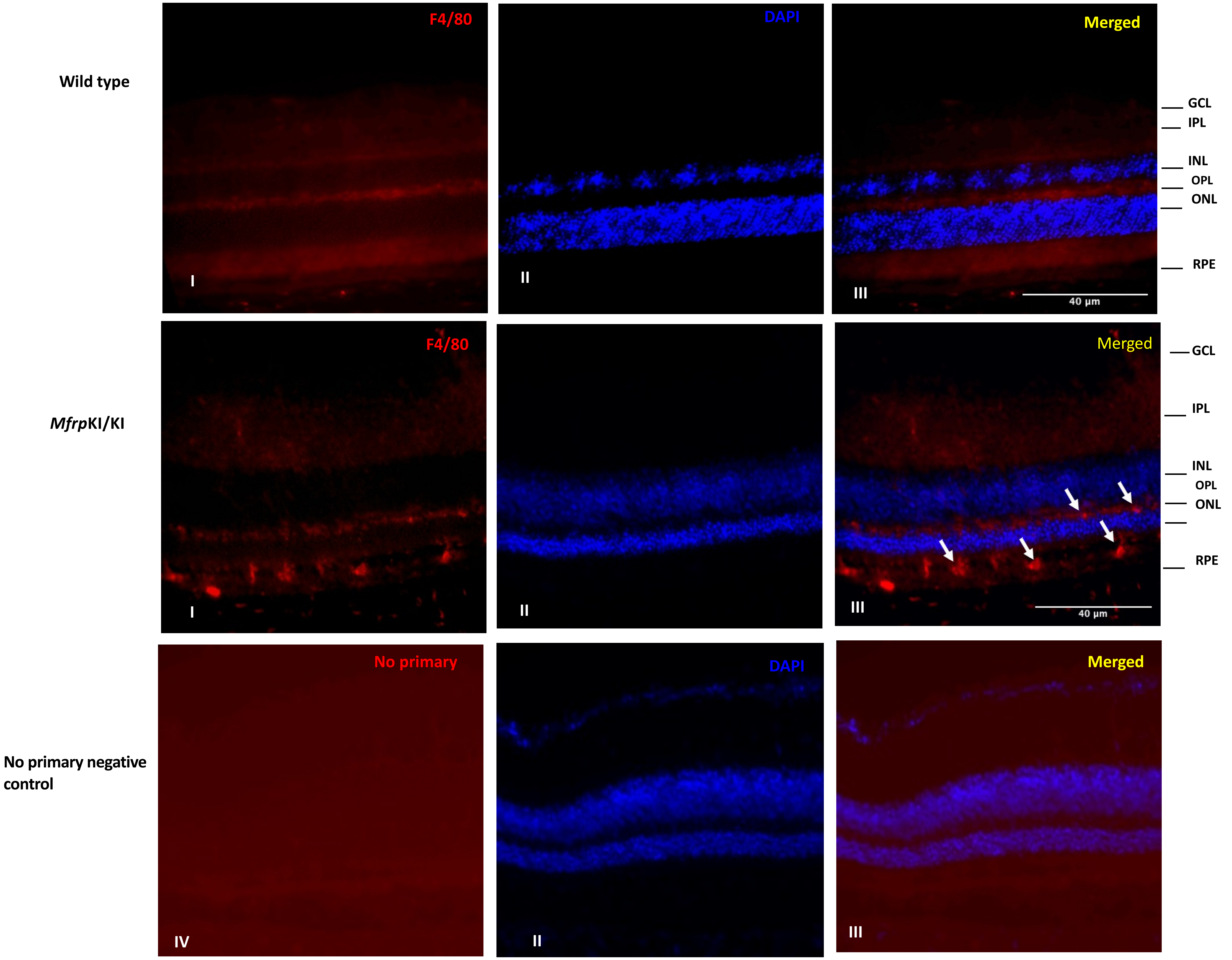
Wild type
F4/80
I
DAPI
II
Merged
III
40 µm
GCL
IPL
INL
OPL
ONL
RPE
*Mfrp*KI/KI
F4/80
I
II
Merged
III
40 µm
GCL
IPL
INL
OPL
ONL
RPE
No primary negative control
No primary
IV
DAPI
II
Merged
III

Supplementary fig 1. Fundus images of three siblings cases carrying c.498_499insC mutation demonstrating a white/crème spot phenotype.

Supplementary fig 2. SD-OCT retinal images of both eyes of a 44 year old male with a history of poor vision carrying compound heterozygote for mutations in the *MFRP* gene c.523C>T, p.(Gln175*) and c.649G>A, p. (Gly217Arg) **(A)** Right and **(B)** Left widefield autofluorescent imaging demonstrating widespread retinal degeneration**, (C)** Right and **(D)** left eye multicolor imaging demonstrates discrete creamy spots near in the macula (white arrowheads).(**E**) SD-OCT image from mice retinal showing autofluorescent white spot.

Supplementary fig 3. (A) Probability density of Mfrp$^{KI/KI}$ and WT microglial cells in multidimensional coordinate space showing higher heterogeneity as compared to WT **(B)** entropy of distribution function of microglial cells from Mfrp $^{KI/KI}$ and WT.

Supplementary fig 4. Phagocytic and Apoptotic pathway associated gene expression differences comparing WT and Mfrp $^{KI/KI}$ retinal microglia.

Supplementary fig 5. Immunostaining of retinal cross sections for F4/80 showing positive cells were significantly greater in *Mfrp* $^{KI/KI}$ mice retina compared with WT retina (I-F4/80, II-DAPI, III-Brightfield (BF) IV-Merged) (*p<0.05)(n=3) and mainly seen in the GCL, IPL (white arrowhead) and INL (white arrows)) GCL -Ganglion Cell layer, IPL-Inner plexiform layer, INL-Inner nuclear layer, OPL-Outer plexiform layer, ONL-Outer plexiform layer, RPE- Retinal Pigment Epithelial cell layer).

Supplementary fig 6. Immunodetection of F4/80 was performed by sodium borohydrite retrieval system. Large prominent F4/80 positive microglia were detected in Mfrp KI/KI retinal section but were absent from WT in any layer (I-F4/80, II-DAPI, III-Merged, IV -no primary negative control). GCL -Ganglion Cell layer, IPL-Inner plexiform layer, INL-Inner nuclear layer, OPL-Outer plexiform layer, ONL-Outer plexiform layer, RPE- Retinal Pigment Epithelial cell layer).

Supplementary table A. Details of the *Mfrp* variants in the new patient with *Mfrp* associated retinal degeneration.

Gene	Mutation	Gnomad (AC/AN)	SIFT	Polyphen	Mutationtaster	Classification
Mfrp	c.523C>T, p.(Gln175*), rs121908189	10/248650 Allele count in Latino/admixed American - 8	N/A	N/A	Disease causing	Pathogenic
Mfrp	c.649G>A, p.(Gly217Arg)	0/0	Deleterious	Probably damaging	Disease causing	Variant of uncertain significance

Supplementary table B. Microglial activation and homeostatic markers (comparison between WT and *Mfrp*$^{KI/KI}$) (*lfdr*<0.1)

xens	xgene	Xsym	m1_0	m2_0	lr	fc	z	lfdr
ENSMUSG00000052684	16476	Jun	0.000741	0.000423	-0.80835	-1.75121	-12.3583	6.66E-14
ENSMUSG00000015852	80891	Fcrls	0.000269	0.000156	-0.79158	-1.73097	-6.71919	0.004172
ENSMUSG00000036362	74191	P2ry13	0.000624	0.00044	-0.50457	-1.4187	-9.79613	6.91E-08
ENSMUSG00000013663	19211	Pten	0.000232	0.000169	-0.45835	-1.37397	-5.49506	0.066298
ENSMUSG00000033713	71375	Foxn3	0.000473	0.000345	-0.45468	-1.37048	-8.00218	9.51E-05
ENSMUSG00000052336	13051	Cx3cr1	0.007087	0.005204	-0.44539	-1.36168	-18.3197	2.60E-33
ENSMUSG00000030671	18576	Pde3b	0.000355	0.00027	-0.39651	-1.31632	-6.05585	0.020088
ENSMUSG00000014361	17289	Mertk	0.00059	0.000454	-0.37867	-1.30014	-6.64975	0.004877
ENSMUSG00000036353	70839	P2ry12	0.001925	0.001482	-0.37764	-1.29921	-10.8448	2.76E-10
ENSMUSG00000031665	58198	Sall1	0.000463	0.000359	-0.36743	-1.29005	-6.39347	0.009322
ENSMUSG00000054675	231633	Tmem119	0.001488	0.001163	-0.35565	-1.27956	-9.20479	8.53E-07
ENSMUSG00000007613	21812	Tgfbr1	0.001317	0.001073	-0.29487	-1.22678	-7.37113	0.000663
ENSMUSG00000051504	233274	Siglech	0.001478	0.001228	-0.2683	-1.20439	-6.84533	0.00289
ENSMUSG00000024397	11629	Aif1/Iba-*1*	0.000603	0.000733	0.282802	1.216556	6.473948	0.000119
ENSMUSG00000018774	12514	Cd68	0.000486	0.000592	0.28345	1.217102	5.250332	0.005017
ENSMUSG00000004730	13733	Adgre1/F4/80	0.000151	0.000216	0.51448	1.428479	5.549719	0.002013
ENSMUSG00000036067	227659	Slc2a6	1.33E-05	2.66E-05	1.006938	2.009641	4.4722	0.035948
ENSMUSG00000042429	11539	Adora1	9.67E-06	2.34E-05	1.274974	2.419945	4.80707	0.015774
ENSMUSG00000062082	239849	Cd200r4	2.65E-06	9.50E-06	1.841555	3.583962	4.132685	0.075027

Supplementary table C. Inflammatory markers after comparison between WT and *Mfrp* $^{KI/KI}$ (*lfdr*<0.1)

xens	xgene	xsym	m1_0	m2_0	lr	fc	z	lfdr
ENSMUSG00000000290	16414	Itgb2	0.000305	0.000372	0.284511	1.217997	4.445511	0.0379
ENSMUSG00000031447	16783	Lamp1	0.00078	0.000982	0.331755	1.258543	8.602265	1.88E-08
ENSMUSG00000022108	16432	Itm2b	0.003139	0.00406	0.370853	1.293117	15.32149	4.31E-27
ENSMUSG00000033307	17319	Mif	8.50E-05	0.000112	0.391923	1.312141	3.480926	0.232503
ENSMUSG00000021109	15251	Hif1a	0.000124	0.000165	0.411452	1.330024	4.283928	0.052288
ENSMUSG00000028692	58810	Akr1a1	0.000179	0.000252	0.494629	1.408959	6.645191	5.42E-05
ENSMUSG00000058818	18733	Pirb	6.43E-05	9.27E-05	0.529116	1.443045	4.111956	0.078143
ENSMUSG00000025351	12512	Cd63	0.000542	0.000782	0.530086	1.444015	9.153959	1.78E-09
ENSMUSG00000018920	66102	Cxcl16	0.000116	0.000169	0.535534	1.449478	4.922832	0.012163
ENSMUSG00000000682	23833	Cd52	0.000587	0.000861	0.552875	1.467006	11.78388	4.35E-16
ENSMUSG00000064351	17708	COX1	0.003704	0.005517	0.574696	1.489364	12.88605	2.02E-19
ENSMUSG00000059089	246256	Fcgr4	6.96E-05	0.000104	0.580888	1.49577	4.777841	0.016686
ENSMUSG00000041736	12257	Tspo	0.000145	0.000223	0.624762	1.541956	7.825009	5.93E-07
ENSMUSG00000027995	24088	Tlr2	0.000116	0.000185	0.678154	1.600091	6.49036	0.000113
ENSMUSG00000015396	12522	Cd83	9.97E-05	0.000164	0.718867	1.645889	5.991996	0.000548
ENSMUSG00000035493	21810	Tgfbi	3.60E-05	6.01E-05	0.739226	1.66928	4.179393	0.068004
ENSMUSG00000031555	11502	Adam9	2.93E-05	4.99E-05	0.767328	1.702115	4.487938	0.034798
ENSMUSG00000040033	20847	Stat2	6.68E-05	0.000116	0.795653	1.735863	5.46248	0.002494
ENSMUSG00000064354	17709	COX2	0.002366	0.004189	0.824278	1.770648	13.94028	1.57E-22
ENSMUSG00000037321	21354	Tap1	5.43E-05	9.75E-05	0.84392	1.794921	6.424056	0.000139
ENSMUSG00000026104	20846	Stat1	0.000115	0.000209	0.854445	1.808063	7.952927	4.25E-07
ENSMUSG00000014599	12977	Csf1	2.53E-05	4.59E-05	0.858439	1.813075	3.909809	0.110887
ENSMUSG00000053835	15042	H2-T24	1.60E-05	3.06E-05	0.931544	1.907316	4.060542	0.085873
ENSMUSG00000021886	14744	Gpr65	5.76E-05	0.000111	0.941948	1.92112	6.360614	0.002
ENSMUSG00000031304	16186	Il2rg	1.98E-05	3.95E-05	0.993743	1.991345	4.710831	0.018776
ENSMUSG00000035352	20293	Ccl12	0.000435	0.000916	1.073303	2.104246	6.561202	8.53E-05
ENSMUSG00000004709	18106	Cd244a	2.19E-05	4.62E-05	1.080394	2.114613	4.961071	0.01097
ENSMUSG00000028459	12517	Cd72	7.02E-05	0.00016	1.183858	2.271835	9.44783	3.42E-10
ENSMUSG00000035385	20296	Ccl2	2.58E-05	6.07E-05	1.231049	2.347376	3.73647	0.156892
ENSMUSG00000030577	12483	Cd22	1.51E-05	3.54E-05	1.234569	2.353111	5.507907	0.002185
ENSMUSG00000030789	16411	Itgax	1.73E-05	4.43E-05	1.355717	2.559242	6.776662	4.01E-05
ENSMUSG00000033467	57914	Crlf2	3.84E-05	0.000108	1.484228	2.797674	10.25496	4.72E-12
ENSMUSG00000024610	16149	Cd74	0.000273	0.000803	1.556305	2.940996	4.88218	0.013431
ENSMUSG00000035042	20304	Ccl5	3.75E-05	0.000119	1.664858	3.170825	4.881381	0.013456
ENSMUSG00000030785	12862	Cox6a2	1.01E-05	3.24E-05	1.683853	3.212847	6.246519	0.000213
ENSMUSG00000024529	16948	Lox	1.31E-05	4.22E-05	1.692079	3.231221	6.489206	0.000113
ENSMUSG00000040612	1E+08	Ildr2	1.21E-05	4.12E-05	1.767651	3.40499	7.092495	1.17E-05

ENSMUSG00000020053	16000	Igf1	7.06E-06	2.86E-05	2.018699	4.052182	5.097158	0.006838

Supplementary table D. Results of cluster 2 and cluster 7 comparison for Homeostatic markers (*lfdr*<0.1)

xens	xgene	xsym	m1_0	m2_0	lr	fc	z	lfdr
ENSMUSG00000015852	80891	Fcrls	0.000241	0.000122	-0.98129	-1.97423	-6.13859	0.008668
ENSMUSG00000090137	22186	Uba52	0.000504	0.000292	-0.78442	-1.7224	-9.56424	1.38E-08
ENSMUSG00000024565	20689	Sall3	0.00028	0.000181	-0.62764	-1.54503	-6.37129	0.004992
ENSMUSG00000036353	70839	P2ry12	0.002161	0.001424	-0.60175	-1.51755	-13.4472	1.07E-18
ENSMUSG00000036362	74191	P2ry13	0.000649	0.000432	-0.58562	-1.50068	-8.79406	5.50E-07
ENSMUSG00000052336	13051	Cx3cr1	0.007608	0.005128	-0.5692	-1.4837	-18.936	2.32E-39
ENSMUSG00000054675	231633	Tmem119	0.001636	0.0012	-0.44686	-1.36307	-9.57798	1.25E-08
ENSMUSG00000014361	17289	Mertk	0.000597	0.000441	-0.43662	-1.35343	-6.28634	0.006334
ENSMUSG00000051504	233274	Siglech	0.001558	0.001225	-0.34609	-1.27111	-7.57065	7.97E-05
ENSMUSG00000007613	21812	Tgfbr1	0.001332	0.001058	-0.3318	-1.25858	-6.80473	0.001245

Supplementary table E. Results of cluster 2 and cluster 7 comparison for Activation markers (*lfdr*<0.1)

xens	xgene	xsym	m1_0	m2_0	lr	fc	z	lfdr
ENSMUSG00000018774	12514	Cd68	0.000474	0.000622	0.39109	1.311384	5.805699	0.000526
ENSMUSG00000004730	13733	Adgre1/F4/80	0.000139	0.000201	0.527843	1.441772	4.84757	0.013281
ENSMUSG00000030124	16768	Lag3	0.000117	0.000189	0.694396	1.618207	5.688479	0.000935
ENSMUSG00000030157	93694	Clec2d	8.44E-05	0.00015	0.833767	1.782333	4.660186	0.022526
ENSMUSG00000027712	11747	Anxa5	4.01E-05	9.27E-05	1.20781	2.309867	6.377212	7.39E-05
ENSMUSG00000022906	80285	Parp9	3.15E-05	7.48E-05	1.248169	2.375398	5.463858	0.001769
ENSMUSG00000026104	20846	Stat1	0.000111	0.000265	1.258196	2.391964	8.691738	2.34E-09
ENSMUSG00000069516	17105	Lyz2	0.000298	0.000797	1.420917	2.677557	15.28593	7.81E-30
ENSMUSG00000033880	19039	Lgals3bp	0.000267	0.000722	1.438015	2.709478	16.34954	4.32E-34
ENSMUSG00000042429	11539	Adora1/F4/80	1.01E-05	3.08E-05	1.604902	3.041751	4.845088	0.013357
ENSMUSG00000030789	16411	Itgax	1.51E-05	5.40E-05	1.837625	3.574212	6.746747	1.69E-05
ENSMUSG00000024675	64380	Ms4a4c	5.84E-06	3.07E-05	2.394316	5.257278	4.468882	0.036504
ENSMUSG00000002985	11816	Apoe	0.000316	0.004054	3.683161	12.84523	16.65418	3.68E-35

Supplementary table F. CD68 expression comparison of cluster 2 with other 19 clusters

zxens	xgene	xsym	m1_0	m2_0	lr	fc	z	lfdr	zxens
ENSMUSG00000018774	12514	CD68	0.000474162	9.70E-06	-5.61124	-48.8823	-9.24766	0.000122	C2 with C1
ENSMUSG00000018774	12514	CD68	0.000474162	1.42E-05	-5.05899	-33.3355	-10.7364	0.000196	C2 with C3
ENSMUSG00000018774	12514	CD68	0.000474162	1.66E-05	-4.8384	-28.609	-7.45107	0.000127	C2 with C4
ENSMUSG00000018774	12514	CD68	0.000474162	0.000619	0.383538	1.304538	0.919079	0.108277	C2 with C5
ENSMUSG00000018774	12514	CD68	0.000474162	0.001323	1.48024	2.789951	7.037823	3.32E-10	C2 with C6
ENSMUSG00000018774	12514	CD68	0.000474162	0.000622	0.39109	1.311384	5.805699	0.000526	C2 with C7
ENSMUSG00000018774	12514	CD68	0.000474162	0.000147	-1.68614	-3.21794	-2.51153	1	C2 with C8
ENSMUSG00000018774	12514	CD68	0.000474162	1.10E-05	-5.43378	-43.2246	-5.76154	0.003718	C2 with C9
ENSMUSG00000018774	12514	CD68	0.000474162	0.000578	0.286241	1.219459	2.72361	0.485942	C2 with C10
ENSMUSG00000018774	12514	CD68	0.000474162	0.000474	0.000109	1.000076	0.000478	1	C2 with C11
ENSMUSG00000018774	12514	CD68	0.000474162	0.000719	0.601219	1.516998	1.636946	1	C2 with C12
ENSMUSG00000018774	12514	CD68	0.000474162	0.000476	0.00479	1.003326	0.036855	1	C2 with C13
ENSMUSG00000018774	12514	CD68	0.000474162	0.00051	0.104036	1.074776	0.949062	1	C2 with C14
ENSMUSG00000018774	12514	CD68	0.000474162	5.89E-06	-6.33094	-80.5013	-9.4401	1.77E-05	C2 with C15
ENSMUSG00000018774	12514	CD68	0.000474162	0.001547	1.705614	3.261677	2.69222	0.001528	C2 with C16
ENSMUSG00000018774	12514	CD68	0.000474162	0	#NAME?	#NAME?	-10.8662	3.16E-05	C2 with C17
ENSMUSG00000018774	12514	CD68	0.000474162	0	#NAME?	#NAME?	-4.22965	3.19E-05	C2 with C18
ENSMUSG00000018774	12514	Cd68	0.000474162	0.000362	-0.38871	-1.30923	-1.29184	1	C2 with C19
ENSMUSG00000018774	12514	Cd68	0.000474162	0.00043	-0.14253	-1.10384	-0.46792	1	C2 with C20

Supplementary table G. Expression of different markers in Cluster 6 and 16 vs Cluster 2

		Cluster 2 vs Cluster 6						
**xens**	**xgene**	**xsym**	**m1_0**	**m2_0**	**lr**	**fc**	**z**	**lfdr**
ENSMUSG00000014361	17289	Mertk	0.0005975	0.0002554	-1.2261304	-2.3393867	-5.1436684	0.005232
ENSMUSG00000036362	74191	P2ry13	0.0006487	0.0002597	-1.3206657	-2.4978134	-5.9209977	0.0001792
ENSMUSG00000054675	231633	Tmem119	0.0016359	0.0006138	-1.4143352	-2.6653689	-7.3322089	1.27E-07
ENSMUSG00000052336	13051	Cx3cr1	0.0076084	0.002642	-1.5259344	-2.8797317	-13.794826	3.18E-33
ENSMUSG00000036353	70839	P2ry12	0.002161	0.0007122	-1.601318	-3.0342038	-9.6938602	1.46E-14
ENSMUSG00000031665	58198	Sall1	0.0005059	0.0001667	-1.6017106	-3.0350295	-5.6707	0.0005773
ENSMUSG00000024672	109225	Ms4a7	2.65E-07	0.0003001	10.144082	1131.5482	3.9278064	0.0056711
ENSMUSG00000069516	17105	Lyz2	0.0002977	0.0023817	2.9998131	7.9989637	9.2904922	5.24E-18
ENSMUSG00000033880	19039	Lgals3bp	0.0002666	0.000648	1.2815503	2.4310007	4.2745205	0.0013148
ENSMUSG00000050335	16854	Lgals3	3.79E-06	0.0004284	6.8191276	112.91768	3.4333505	0.029747
ENSMUSG00000053063	-10534.4	Clec12a	0.0001711	2.16E-03	Inf	Inf	2.3250793	0.0011224
ENSMUSG00000002985	11816	Apoe	0.0003156	0.033529	6.7311536	106.23782	10.353199	2.64E-22
ENSMUSG00000018774	12514	Cd68	0.0004742	0.0013229	1.4802399	2.7899512	7.0378233	3.32E-10
ENSMUSG00000024397	11629	Aif1	0.0006592	0.0008115	0.2999666	1.2311159	1.5900315	0.7713315
ENSMUSG00000004730	13733	Adgre1	0.0001395	0.0002464	0.8207582	1.766334	2.6326839	0.2089586
		**Cluster 2 vs Cluster 16**						
**xens**	**xgene**	**xsym**	**m1_0**	**m2_0**	**lr**	**fc**	**z**	**lfdr**
ENSMUSG00000054675	231633	Tmem119	0.0016359	0.0004948	-1.7251437	-3.3061306	-3.5169753	0.0243749
ENSMUSG00000052336	13051	Cx3cr1	0.0076084	0.0018365	-2.0505897	-4.1427526	-7.4685167	1.47E-16
ENSMUSG00000036353	70839	P2ry12	0.002161	0.0003014	-2.8420945	-7.1706031	-5.7880759	3.39E-09
ENSMUSG00000069516	17105	Lyz2	0.0002977	0.0026295	3.1426519	8.8314597	2.2620832	0.0084265
ENSMUSG00000073421	14961	H2-Ab1	3.59E-05	0.0002512	2.8072404	6.9994441	1.5327802	0.0526994
ENSMUSG00000024675	64380	Ms4a4c	5.84E-06	0.0004702	6.3301559	80.45755	1.7161584	0.0359231
ENSMUSG00000053063	232413	Clec12a	0	0.0004731	Inf	Inf	2.2945762	0.0075738
ENSMUSG00000018774	12514	Cd68	0.0004742	0.0015466	1.7056138	3.2616766	2.6922198	0.0015281
ENSMUSG00000024397	11629	Aif1	0.0006592	0.00019	-1.7948775	-3.4698601	-2.9548332	0.2878091
ENSMUSG00000004730	13733	Adgre1	0.0001395	0.0003676	1.3980677	2.6354836	1.415213	0.0660844

Supplementary table H. Expression of different markers in cluster 10, 13 and 14 vs cluster

			**Cluster 2 vs Cluster 10**					
**Xens**	**xgene**	**xsym**	**m1_0**	**m2_0**	**lr**	**fc**	**z**	**lfdr**
ENSMUSG00000036353	70839	P2ry12	0.0021611	0.00112497	-0.9418445	-1.9209827	-13.627076	1.00E-24
ENSMUSG00000054675	231633	Tmem119	0.0016359	0.00080852	-1.0167375	-2.0233382	-13.109863	1.51E-22
ENSMUSG00000036362	74191	P2ry13	0.0006487	0.0003703	-0.8088155	-1.7517726	-8.2093477	9.10E-08
ENSMUSG00000002985	11816	Apoe	0.0003156	0.02219273	6.1358326	70.31851	17.93526	3.43E-50
ENSMUSG00000069516	17105	Lyz2	0.0002978	0.00177238	2.5735344	5.9526597	12.845637	7.13E-26
ENSMUSG00000025351	12512	Cd63	0.000465	0.00087962	0.919589	1.8915763	8.4631583	6.70E-11
ENSMUSG00000033880	19039	Lgals3bp	0.0002666	0.00050007	0.9076315	1.8759632	6.7362528	7.98E-07
ENSMUSG00000024672	109225	Ms4a7	2.65E-07	0.00046792	10.784892	1764.3148	7.6742529	5.88E-09
ENSMUSG00000079419	73656	Ms4a6c	5.60E-05	0.00020473	1.8712289	3.6584407	7.2253773	6.13E-08
ENSMUSG00000015396	12522	Cd83	8.52E-05	0.00016929	0.9900667	1.9862768	4.5149454	0.0121813
ENSMUSG00000024610	16149	Cd74	2.28E-05	0.0007271	4.9966823	31.926496	5.9108121	3.56E-05
ENSMUSG00000018774	12514	Cd68	0.0004742	0.00057822	0.2862411	1.2194589	2.7236097	0.4859415
ENSMUSG00000024397	11629	Aif1	0.0006592	0.00070481	0.0965956	1.0692473	1.1346279	0.9951912
ENSMUSG00000004730	13733	Adgre1	0.0001395	0.00023176	0.7326331	1.6616691	4.5994349	0.0091691
			**Cluster 2 vs Cluster 13**					
**xens**	**xgene**	**xsym**	**m1_0**	**m2_0**	**lr**	**fc**	**z**	**lfdr**
ENSMUSG00000052336	13051	Cx3cr1	0.0076084	0.00630999	-0.2699461	-1.2057628	-5.7798987	0.0008737
ENSMUSG00000054675	231633	Tmem119	0.0016359	0.00125546	-0.3818838	-1.3030422	-4.7978214	0.0237652
ENSMUSG00000036353	74191	P2ry12	0.0021611	0.00178214	-0.2781216	-1.212615	-4.1448062	0.1087197
ENSMUSG00000033880	19039	Lgals3bp	0.0002666	0.00029943	0.1677143	1.1232774	0.9348465	1
ENSMUSG00000024610	16149	Cd74	2.28E-05	2.56E-05	0.1673826	1.1230192	0.3404226	1
ENSMUSG00000024672	109225	Ms4a7	2.65E-07	2.54E-05	6.5790764	95.609126	2.3860739	1
ENSMUSG00000024397	11629	Aif1	0.0006592	0.0005133	-0.3608338	-1.2841679	-3.3603778	0.3434475
ENSMUSG00000018774	12514	Cd68	0.0004742	0.00047574	0.0047901	1.0033258	0.0368555	1
ENSMUSG00000004730	13733	Adgre1	0.0001395	0.000185	0.407529	1.326412	1.9357221	1
			**Cluster 2 vs Cluster 14**					
**xens**	**xgene**	**xsym**	**m1_0**	**m2_0**	**lr**	**fc**	**z**	**lfdr**

ENSMUSG00000069516	17105	Lyz2	0.0002978	0.00073045	1.2946953	2.4532517	9.9784671	1.00E-18
ENSMUSG00000002985	11816	Apoe	0.0003156	0.00149396	2.2429574	4.7336644	8.9392532	4.81E-15
ENSMUSG00000033880	19039	Lgals3bp	0.0002666	0.00041466	0.6374181	1.5555428	4.2638377	0.0090106
ENSMUSG00000015852	80891	Fcrls	0.0002412	9.87E-05	-1.2886069	-2.4429205	-5.1925108	0.0146152
ENSMUSG00000015852	80891	Fcrls	0.0002412	9.87E-05	-1.2886069	-2.4429205	-5.1925108	0.0146152
ENSMUSG00000018774	12514	Cd68	0.0004742	0.00050962	0.1040357	1.0747757	0.9490624	1
ENSMUSG00000004730	13733	Adgre1	0.0001395	0.00015942	0.1927891	1.1429712	1.1391102	1
ENSMUSG00000024397	11629	Aif1	0.0006592	0.00087704	0.4119978	1.330527	5.1111952	0.0002476

Supplementary table I. Results of cluster 7 and cluster 6 comparison (*lfdr*<0.1)

xens	xgene	xsym	m1_0	m2_0	lr	fc	z	lfdr
ENSMUSG00000024672	109225	Ms4a7	0.0003	2.11E-05	-3.83094	-14.2307	-3.43217	0.059701
ENSMUSG00000002985	11816	Apoe	0.033529	0.004054	-3.04799	-8.27061	-9.14234	2.20E-17
ENSMUSG00000027712	11747	Anxa5	0.000295	9.27E-05	-1.66943	-3.18089	-3.42579	0.060839
ENSMUSG00000015852	80891	Fcrls	0.00038	0.000122	-1.63783	-3.11198	-3.81505	0.017452
ENSMUSG00000069516	17105	Lyz2	0.002382	0.000797	-1.5789	-2.98741	-6.47178	3.72E-08
ENSMUSG00000025351	12512	Cd63	0.002072	0.000821	-1.33568	-2.52395	-7.7792	9.12E-12
ENSMUSG00000018774	12514	Cd68	0.001323	0.000622	-1.08915	-2.12749	-5.51956	1.23E-05
ENSMUSG00000060126	22070	Tpt1	0.004507	0.003187	-0.50019	-1.4144	-4.48879	0.001734
ENSMUSG00000024621	12978	Csf1r	0.001934	0.002716	0.489562	1.404018	4.432788	0.041987
ENSMUSG00000021423	17084	Ly86	0.00196	0.003073	0.648858	1.567927	5.049504	0.005018
ENSMUSG00000022587	17069	Ly6e	0.001194	0.001907	0.675362	1.596997	4.265414	0.069853
ENSMUSG00000018593	20692	Sparc	0.000943	0.001739	0.883254	1.844531	5.083006	0.004414
ENSMUSG00000052336	13051	Cx3cr1	0.002642	0.005128	0.956739	1.940918	7.355927	4.01E-08
ENSMUSG00000054675	231633	Tmem119	0.000614	0.0012	0.967477	1.955418	4.313535	0.061133
ENSMUSG00000036353	70839	P2ry12	0.000712	0.001424	0.999571	1.999405	5.046685	0.005069
ENSMUSG00000051504	233274	Siglech	0.000416	0.001225	1.559707	2.94794	7.273792	8.16E-08
ENSMUSG00000079227	12774	Ccr5	7.83E-05	0.000296	1.915754	3.77311	4.329496	0.05824

Supplementary table J. Gene expression showing comparison between cluster 8 and 15 (*lfdr*<0.1) Activation markers

xens	xgene	xsym	m1_0	m2_0	lr	fc	z	lfdr
ENSMUSG00000018593	20692	Sparc	0.001993	0	#NAME?	#NAME?	-9.56135	1.24E-30
ENSMUSG00000027435	17064	Cd93	0	0.000368	Inf	Inf	5.052882	6.06E-11
ENSMUSG00000035373	20306	Ccl7	0	0.000111	Inf	Inf	3.873516	1.42E-06

ENSMUSG00000008845	93671	Cd163	0	0.000471	Inf	Inf	3.808476	2.71E-06
ENSMUSG00000068220	16852	Lgals1	1.12E-07	0.000198	10.7898	1770.321	4.032583	4.24E-07
ENSMUSG00000024610	16149	Cd74	2.32E-05	0.003877	7.38592	167.2567	4.510896	9.40E-09
ENSMUSG00000036594	14960	H2-Aa	8.02E-06	0.001285	7.323705	160.1972	4.160346	1.81E-07
ENSMUSG00000029084	12494	Cd38	2.27E-06	0.000297	7.035428	131.1822	5.521732	3.51E-13
ENSMUSG00000025804	12768	Ccr1	4.65E-06	0.00048	6.689343	103.2031	6.308136	6.35E-17
ENSMUSG00000024672	109225	Ms4a7	2.12E-05	0.0009371	5.4658713	44.196841	6.499442822	6.62E-18
ENSMUSG00000073421	14961	H2-Ab1	3.35E-05	0.001349	5.33113	40.25595	3.419898	4.15E-05
ENSMUSG00000002985	11816	Apoe	0.001398	0.053743	5.265057	38.45388	10.40147	2.77E-45
ENSMUSG00000053063	232413	Clec12a	3.39E-06	0.0001188	5.1291807	34.997518	2.316990074	0.0160658
ENSMUSG00000027009	16401	Itga4	7.52E-06	0.000248	5.042666	32.96049	3.020524	0.000485
ENSMUSG00000069516	17105	Lyz2	0.000419	0.007987	4.253974	19.0798	11.34728	8.06E-54
ENSMUSG00000073418	12268	C4b	8.23E-05	0.001413	4.102015	17.17234	7.304535	2.99E-22
ENSMUSG00000035352	20293	Ccl12	0.000695	0.002353	1.759488	3.385779	2.952767	0.000656
ENSMUSG00000040552	12267	C3ar1	0.00025	0.000786	1.651457	3.141508	4.435541	1.48E-08
ENSMUSG00000004730	13733	Adgre1/F4/80	0.000186	0.000582	1.646375	3.13046	4.0047	5.98E-07
ENSMUSG00000060126	22070	Tpt1	0.002235	0.006466	1.532916	2.893701	8.50846	3.24E-30
ENSMUSG00000041736	12257	Tspo	0.00012	0.000325	1.439959	2.713131	2.434457	0.009539
ENSMUSG00000049130	12273	C5ar1	0.000148	0.000321	1.113548	2.163771	2.36066	0.012865

Supplementary table K. Gene expression showing comparison between cluster 8 and 15 (*lfdr*<0.1) (Homeostatic markers).

xens	xgene	xsym	m1_0	m2_0	lr	fc	z	lfdr
ENSMUSG00000031665	58198	Sall1	0.000483	5.21E-06	-6.53635	-92.819	-5.48601	1.75E-08
ENSMUSG00000054675	231633	Tmem119	0.001401	3.92E-05	-5.16095	-35.7768	-7.8185	2.42E-19
ENSMUSG00000032010	53376	Usp2	0.000174	6.28E-06	-4.79083	-27.6811	-3.63473	0.014362
ENSMUSG00000036353	70839	P2ry12	0.001944	0.000191	-3.34529	-10.1633	-8.14353	4.12E-21
ENSMUSG00000007613	21812	Tgfbr1	0.001389	0.000143	-3.28515	-9.7483	-7.26925	2.06E-16
ENSMUSG00000040229	23890	Gpr34	0.001472	0.000156	-3.2373	-9.43029	-6.63143	2.86E-13
ENSMUSG00000035133	11855	Arhgap5	0.001382	0.000168	-3.03741	-8.21018	-6.48261	1.07E-12
ENSMUSG00000014361	17289	Mertk	0.000616	7.73E-05	-2.99493	-7.97195	-4.55104	4.32E-05
ENSMUSG00000036362	74191	P2ry13	0.00055	9.74E-05	-2.49848	-5.65089	-4.11438	0.000821
ENSMUSG00000051504	233274	Siglech	0.001482	0.000444	-1.74052	-3.34155	-5.23854	1.35E-07
ENSMUSG00000052336	13051	Cx3cr1	0.006644	0.002127	-1.64328	-3.12375	-7.43666	2.57E-17

Supplementary table L. Phagocytic and Apoptotic pathway associated gene expression as a result of comparison between WT and *Mfrp* $^{KI/KI}$ (*lfdr*<0.1)

xens	xgene	xsym	m1_0	m2_0	lr	fc	z	lfdr
ENSMUSG00000038642	13040	Ctss	0.004924	0.006084108	0.305105	1.235508	11.97111	1.69E-16
ENSMUSG00000021477	13039	Ctsl	0.001245	0.001565748	0.331199	1.258059	8.432579	5.56E-08
ENSMUSG00000005881	66366	Ergic3	0.000126	0.000163759	0.375486	1.297276	4.207332	0.063804
ENSMUSG00000096727	16912	Psmb9	7.81E-05	0.000107983	0.467152	1.382377	4.294547	0.050691
ENSMUSG00000028692	58810	Akr1a1	0.000179	0.000251794	0.494629	1.408959	6.645191	5.42E-05
ENSMUSG00000021242	67963	Npc2	0.000652	0.000934954	0.520562	1.434514	13.17474	3.91E-20
ENSMUSG00000034422	547253	Parp14	0.000189	0.000274755	0.539016	1.452981	5.009316	0.009464
ENSMUSG00000021939	13030	Ctsb	0.001711	0.002500289	0.54719	1.461237	14.82184	1.20E-25
ENSMUSG00000041736	12257	Tspo	0.000145	0.000223399	0.624762	1.541956	7.825009	5.93E-07
ENSMUSG00000064345	17717	ND2	0.001226	0.001937563	0.660269	1.580377	9.746775	5.56E-11
ENSMUSG00000016427	54405	Ndufa1	6.73E-05	0.000107661	0.678674	1.600668	6.173198	0.00031
ENSMUSG00000064370	17711	CYTB	0.001812	0.002906076	0.68128	1.603562	11.90061	2.69E-16
ENSMUSG00000022906	80285	Parp9	3.28E-05	5.81E-05	0.82322	1.769351	4.477761	0.035542
ENSMUSG00000064360	17718	ND3	0.000933	0.001670711	0.839802	1.789804	13.93644	1.65E-22
ENSMUSG00000037321	21354	Tap1	5.43E-05	9.75E-05	0.84392	1.794921	6.424056	0.000139
ENSMUSG00000027712	11747	Anxa5	5.22E-05	9.41E-05	0.850259	1.802824	6.166348	0.000319

Table Abbreviations –
Xens= Ensemble gene ID, xgene = Gene ID ,xsym = Gene symbol, m1_0 = mean expression in sample A, m2_0 = mean expression in sample B, lr = log2-ratio:log2(m2_0/ m1_0), Fc= log 2 Fold change, Z = z-score, lfdr = Local false discovery rate

Analytical codes used in ScRNA sequencing analysis

```
load("../P.RData") #P,sym,gene,ens,nb
#3404x18727
load("../res_cir_cl16.RData") #res,cir,cl16,nb

#-----------------------begin clustering/heatmap
stuff-------------------------------
library(cba)

hclust.olo<-function(d, method) {
   hc<-hclust(d, method=method)
   co<-order.optimal(d,hc$merge)
   hc$merge<-co$merge
   hc$order<-co$order
   return(hc)
}

#define color scale
n<-100
r<-(0:n)/n
g<-r
b<-pmin(r+.75,1)
col1<-rgb(r,g,b)
col2<-rev(rgb(b,g,r))
colors<-c(col1,col2[-1])
colors[n+1]<-"#D0D0D0" #grey
#-----------------------end clustering/heatmap
stuff-------------------------------



mS<-colMeans(P)
detected<-mS>=0.000005
n<-sum(detected)
# 10573 genes

x<-P[,detected]
xsym<-sym[detected]
xgene<-gene[detected]
xens<-ens[detected]

cnb<-cumsum(nb)
ns<-length(nb)
ncell<-cnb[ns]

library(locfdr) #works
#do resWT_resKO first
comparison<-"resWT_resKO"
indx1<-intersect(res,1:cnb[1])
x1<-t(x[indx1,]) #1165 cells
```

```
indx2<-intersect(res,(cnb[1]+1):cnb[2])
x2<-t(x[indx2,]) #1784 cells
n1<-dim(x1)[2]
n2<-dim(x2)[2]

#SCRAMBLE CELLS in each condition
set.seed(2357)
x1<-x1[,sample(n1)]
x2<-x2[,sample(n2)]

x0<-cbind(x1,x2)

m1_0<-rowMeans(x1)
m2_0<-rowMeans(x2)


#heatmaps
for (gs in 3:5) {
   X<-
read.table(paste0("resWT_resKO_heatmap_",gs,"_genes.txt.csv"),sep="\t"
,header=TRUE,stringsAsFactors=FALSE)

   sym<-X[,3]
   good<-!duplicated(sym)
   X<-X[good,]

   ix<-match(X[,1],rownames(x0))
   data<-x0[ix,]
   rownames(data)<-X[,3]
   m<-(m1_0[ix]+m2_0[ix])/2
   ldata<-log2(data/m)
   ldata[ldata== -Inf]<- 0 #cells with count 0 will be colored grey

   filename<-paste0("resWT_resKO_heatmap_",gs)
   maxlr<-2
   data<-ldata
   if (is.vector(data)) return(NULL)

   cat(filename,dim(data),"\n")
   nc<-ncol(data)
   nr<-nrow(data)
   colnames(data)<-rep("",nc)
   if (nr>2) { #do olo, otherwise not
      #try a smoothed-out distance using means only
      #d<-dist(data)
      y<-cbind(rowMeans(data[,1:n1],na.rm=TRUE),rowMeans(data[,-
(1:n1)],na.rm=TRUE))
      d<-dist(y)
      hc<-hclust.olo(d,method="ward.D")
      data<-data[hc$order,] #cluster genes, leave samples as is
```

```
   }

   #prepare data for visualization by flooring and scaling
   data[data>maxlr]<-maxlr
   data[data< -maxlr]<- -maxlr
   data<-(data/maxlr)
   #data[data>0]<-data[data>0]^gamma
   #data[data<0]<- -(-data[data<0])^gamma

   rowh<-0.2
   #set 12 in to be the width of heatmap
   #xyratio<-3
   xyratio<-12/0.2/nc

pdf(paste0(filename,".pdf"),width=nc*rowh*xyratio+1+2,height=nr*rowh+1
+2) #leaves 1+2in blank space on all sides of the color map
   par(mai = c(1, 1, 2, 2)) #starts from bottom, clockwise
   image(1:nc, 1:nr, t(data), xlim = 0.5+ c(0, nc), ylim = 0.5+ c(0,
nr),axes=FALSE,xlab="",ylab="",col=colors)
   lines(c(n1,n1),c(0,nr+1/2),lwd=2)
   labRow<-rownames(data)
   axis(4,1:nr,labels=labRow,las=2,line=0,tick=0)

   #mtext(filename,side=3,line=0,cex=2,font=2,adj=0) #add title
   dev.off()

}
```

```
samples<-c("WT","MFRP")

nb<-rep(0,2) #numbers of bar codes per condition
C<-NULL #initialize empty matrix of counts

for (i in 1:2) {
   sname<-samples[i]
   features<-read.table(paste("/mnt/data1/rsasik/20191001_shyamanga/
results/",sname,"/outs/filtered_feature_bc_matrix/
features.tsv",sep=""),header=FALSE,stringsAsFactors=FALSE)
   ng<-dim(features)[1]
   barcodes<-read.table(paste("/mnt/data1/rsasik/20191001_shyamanga/
results/",sname,"/outs/filtered_feature_bc_matrix/
barcodes.tsv",sep=""),header=FALSE)
   M<-read.table(paste("/mnt/data1/rsasik/20191001_shyamanga/
results/",sname,"/outs/filtered_feature_bc_matrix/
matrix.mtx",sep=""),header=FALSE,skip=3,sep=" ")
   nb[i]<-dim(barcodes)[1] #2741
   counts<-matrix(0,nrow=ng,ncol=nb[i])
   for (j in 1:nb[i]) {
   if (j %% 100 == 0) cat(i,j,"\n")
      good<-M[,2]==j
      counts[M[good,1],j]<-M[good,3]
   }
   C<-cbind(C,counts)
}

rownames(C)<-features[,1]
save(C,nb,file="C.RData")
expressed<-apply(C>0,1,any)
nb2<-nb

library(entropy)
Ecounts<-C[expressed,]
E<-rep(0,sum(nb))
E<-apply(Ecounts,2,entropy)
P<-t(Ecounts)/apply(Ecounts,2,sum) #normalize and transform. Samples
in rows

ens<-colnames(P)
library(mygene) #better annotation
q<-queryMany(ens,scopes="ensembl.gene")
good<-!duplicated(q$query)
uq<-q[good,c(1,4,6,5)]
sym<-uq$symbol
gene<-uq$entrezgene
save(P,sym,gene,ens,nb,file="P.RData")


#pairwise distances, Jensen-Shannon distance
```

```
D<-matrix(0,ncol=nb,nrow=nb)
for (i in 1:(nb-1)) {
   cat(i,"\n")
   for (j in (i+1):nb) {
      D[i,j]<-sqrt(entropy((P[i,]+P[j,])/2)-E[i]/2-E[j]/2)
   }
}
D<-D+t(D)

save(D,file="D.RData")
pdf("D.pdf")

hist(D[upper.tri(D)],breaks=1000,border=NA,col="gray20",xlab=expressio
n(D[ij]),main="")
dev.off()

library(apcluster)
apres<-apcluster(s=-D,q=0) #q should be (0,1) but it's not sensitive
for these data
apres
#there are 20 clusters!
#find % from WT and MFRP
nc<-length(apres@clusters)
wtfraction<-rep(0,nc)
height<-matrix(0,nrow=2,ncol=nc)
for (i in 1:nc) {
   wtfraction[i]<-mean(apres@clusters[[i]]<=nb[1])
   height[1,i]<-sum(apres@clusters[[i]]<=nb[1])
   height[2,i]<-sum(apres@clusters[[i]]>nb[1])
}
save(apres,wtfraction,height,file="apres.RData")

pdf("cluster_mass.pdf")
barplot(height,names.arg=1:nc,cex.names=0.8)
dev.off()



#for plotting, t-SNE dimensional reduction
library(Rtsne)
set.seed(2357)
tsne<-Rtsne(D,is_distance=TRUE,perplexity=30)
save(tsne,file="tsne.RData")
rx<-range(tsne$Y[,1])
ry<-range(tsne$Y[,2])

pdf("tsne.pdf")
plot(tsne$Y,pch=19,cex=.2,xlab="t-SNE 1",ylab="t-SNE
2",main="WT+MFRP",xlim=rx,ylim=ry)
plot(tsne$Y[1:nb[1],],pch=19,cex=.2,xlab="t-SNE 1",ylab="t-SNE
```

```
2",main="WT",xlim=rx,ylim=ry)
plot(tsne$Y[-(1:nb[1]),],pch=19,cex=.2,xlab="t-SNE 1",ylab="t-SNE
2",main="MFRP",xlim=rx,ylim=ry)
dev.off()
pdf("tsne_clusters.pdf")
for (i in 1:nc) {
plot(tsne$Y,pch=19,cex=.2,xlab="t-SNE 1",ylab="t-SNE
2",xlim=rx,ylim=ry)
points(tsne$Y[intersect(apres@clusters[[i]],1:nb[1]),],pch=21,cex=0.5,
bg="cyan",lwd=0.1)
points(tsne$Y[setdiff(apres@clusters[[i]],1:nb[1]),],pch=21,cex=0.5,bg
="red",lwd=0.1)
}
dev.off()
plot(apres,tsne$Y)

#do this for
word<-"resident"
word<-"activated"
word<-"circulating"

#load blue genes that are markers of resident microglia
M<-read.table(paste0("/mnt/data1/rsasik/20191001_shyamanga/
markers/",word,"_genes.txt"),sep="\t",header=TRUE,stringsAsFactors=FAL
SE)
n.set<-dim(M)[1]
heat.WT<-matrix(0,nrow=n.set,ncol=nc)
rownames(heat.WT)<-M$symbol
heat.KO<-heat.WT
gamma<-1. #transformation
in.set<-sym %in% M$symbol
is.expressed<-M$symbol %in% sym
pdf(paste0(word,"_genes.pdf"))
par(mfrow=c(2,1))
for (genesym in M$symbol) {
   cl.expr.WT<-list()
   cl.expr.KO<-list()
   if (genesym %in% sym) {#it is expressed, can plot expression
           l<-match(genesym,sym)
      for (cl in 1:nc) {
              cl.expr.WT[[cl]]<-
P[intersect(apres@clusters[[cl]],1:nb[1]),l]
              cl.expr.KO[[cl]]<-P[intersect(apres@clusters[[cl]],
(nb[1]+1):(nb[1]+nb[2])),l]
      }
      ylim=max(unlist(cl.expr.WT),unlist(cl.expr.KO))

boxplot(cl.expr.WT,cex=0.5,boxwex=0.6,cex.axis=0.7,main=paste0(genesym
,", WT"),xlab="cluster",ylab="expression frequency",ylim=c(0,ylim))
```

```
boxplot(cl.expr.KO,cex=0.5,boxwex=0.6,cex.axis=0.7,main=paste0(genesym
,", KO"),xlab="cluster",ylab="expression frequency",ylim=c(0,ylim))
      heat.WT[genesym,]<-sapply(cl.expr.WT,median)
      heat.KO[genesym,]<-sapply(cl.expr.KO,median)
   }
}
dev.off()

mh<-max(c(heat.WT,heat.KO))
heat.WT<-(heat.WT/mh)
heat.KO<-(heat.KO/mh)

blue<-M$color=="blue"
n.blue<-sum(blue)

red<-M$color=="red"
n.red<-sum(red)

black<-M$color=="black"
n.black<-sum(black)

rowh<-0.2 #rowheight in in
xyratio<-1

pdf(paste0(word,"_blue_heat.pdf"),width=nc*rowh*xyratio+1+2,height=n.b
lue*rowh+1+2) #leaves 1+2in blank space on all sides of the color map
par(mai = c(1, 1, 2, 2)) #starts from bottom, clockwise

image(1:nc, 1:n.blue, t(heat.WT[blue,]), xlim = 0.5+ c(0, nc), ylim =
0.5+ c(0, n.blue),axes=FALSE,xlab="",ylab="",col=rev(col1),main="WT")
#white->blue
labCol<-colnames(heat.WT)
labRow<-rownames(heat.WT[blue,])
axis(3,1:nc,labels=labCol,las=2,line=0,tick=0)
axis(4,1:n.blue,labels=labRow,las=2,line=0,tick=0)
box()

image(1:nc, 1:n.blue, t(heat.KO[blue,]), xlim = 0.5+ c(0, nc), ylim =
0.5+ c(0, n.blue),axes=FALSE,xlab="",ylab="",col=rev(col1),main="KO")
#white->blue
labCol<-colnames(heat.KO)
labRow<-rownames(heat.KO[blue,])
axis(3,1:nc,labels=labCol,las=2,line=0,tick=0)
axis(4,1:n.blue,labels=labRow,las=2,line=0,tick=0)
box()

dev.off()

pdf(paste0(word,"_red_heat.pdf"),width=nc*rowh*xyratio+1+2,height=n.re
d*rowh+1+2) #leaves 1+2in blank space on all sides of the color map
```

```
par(mai = c(1, 1, 2, 2)) #starts from bottom, clockwise

image(1:nc, 1:n.red, t(heat.WT[red,]), xlim = 0.5+ c(0, nc), ylim =
0.5+ c(0, n.red),axes=FALSE,xlab="",ylab="",col=col2,main="WT")
#white->red
labCol<-colnames(heat.WT)
labRow<-rownames(heat.WT[red,])
axis(3,1:nc,labels=labCol,las=2,line=0,tick=0)
axis(4,1:n.red,labels=labRow,las=2,line=0,tick=0)
box()

image(1:nc, 1:n.red, t(heat.KO[red,]), xlim = 0.5+ c(0, nc), ylim =
0.5+ c(0, n.red),axes=FALSE,xlab="",ylab="",col=col2,main="KO")
#white->red
labCol<-colnames(heat.KO)
labRow<-rownames(heat.KO[red,])
axis(3,1:nc,labels=labCol,las=2,line=0,tick=0)
axis(4,1:n.red,labels=labRow,las=2,line=0,tick=0)
box()

dev.off()


pdf(paste0(word,"_black_heat.pdf"),width=nc*rowh*xyratio+1+2,height=n.
black*rowh+1+2) #leaves 1+2in blank space on all sides of the color
map
par(mai = c(1, 1, 2, 2)) #starts from bottom, clockwise

image(1:nc, 1:n.black, t(heat.WT[black,]), xlim = 0.5+ c(0, nc), ylim
= 0.5+ c(0, n.black),axes=FALSE,xlab="",ylab="",col=col3,main="WT")
#white->black
labCol<-colnames(heat.WT)
labRow<-rownames(heat.WT[black,])
axis(3,1:nc,labels=labCol,las=2,line=0,tick=0)
axis(4,1:n.black,labels=labRow,las=2,line=0,tick=0)
box()

image(1:nc, 1:n.black, t(heat.KO[black,]), xlim = 0.5+ c(0, nc), ylim
= 0.5+ c(0, n.black),axes=FALSE,xlab="",ylab="",col=col3,main="KO")
#white->black
labCol<-colnames(heat.KO)
labRow<-rownames(heat.KO[black,])
axis(3,1:nc,labels=labCol,las=2,line=0,tick=0)
axis(4,1:n.black,labels=labRow,las=2,line=0,tick=0)
box()

dev.off()


#export resident supercluster and circulating cluster, plus the 16
```

```
cluster
res<-c()
for (i in c(2,5,6,7,8,10:14,16,20)) res<-c(res,apres@clusters[[i]])
res<-unname(res) #2949 cells

cir<-unname(apres@clusters[[19]]) #circulating macs

cl16<-unname(apres@clusters[[16]]) #the cluster 16
save(res,cir,cl16,nb,file="res_cir_cl16.RData")
```

```
library(slingshot)
library(BiocParallel)
library(clusterExperiment)
library(scone)
library(zinbwave)
library(doParallel)
library(gam)
library(RColorBrewer)

library(cba)
hclust.olo<-function(d, method) {
   hc<-hclust(d, method=method)
   co<-order.optimal(d,hc$merge)
   hc$merge<-co$merge
   hc$order<-co$order
   return(hc)
}

#define colors for heatmaps
ncol<-100
r<-(0:ncol)/ncol
g<-r
b<-pmin(r+.75,1)
col1<-rgb(r,g,b) # red->white
col2<-rev(rgb(b,g,r)) # white->blue
col3<-rev(rgb(r,r,r)) # white->black
colors<-c(col1,col2[-1]) #100 beautiful colors


#need to create an experimental object
samples<-c("WT","MFRP")
ns<-length(samples)
#load distributions
load("/mnt/data1/rsasik/20191001_shyamanga/P.RData")
#P,sym,gene,ens,nb
load("/mnt/data1/rsasik/20191001_shyamanga/C.RData") #C
P<-P[-(1:nb[1]),] #just MFRP MFRP MFRP
ncell<-nb[2]

#need to find which MFRP cluster corresponds to original cluster 2
(WT+MFRP)
#load WT+MFRP clustering
#save(apres,wtfraction,height,file="apres.RData")
load("/mnt/data1/rsasik/20191001_shyamanga/apres.RData")
c_apres<-apres #c_ for combined WT+MFRP
c_nc<-length(apres@clusters)
#load old apres clustering of MFRP data
load("/mnt/data1/rsasik/20191001_shyamanga/apres_MFRP.RData")
MFRP_apres<-apres
nc<-length(apres@clusters)
```

```
mass<-rep(0,nc)
perc<-rep(0,nc)
MFRP_cluster<-rep(0,ncell)
for (i in 1:nc) {
   MFRP_cluster[apres@clusters[[i]]]<-i
   mass[i]<-length(intersect(c_apres@clusters[[2]]-
nb[1],apres@clusters[[i]]))
   perc[i]<-mass[i]/length(apres@clusters[[i]])
}
pdf("MFRP_clusters_in_combined_cluster2.pdf")
   barplot(mass,names=1:nc,ylab="MFRP cells in combined cluster
2",xlab="MFRP cluster")
   barplot(perc,names=1:nc,ylab="fraction of combined cluster 2
cells",xlab="MFRP cluster",ylim=c(0,.5))
dev.off()
#looks like MFRP cluster 8 is the best candidate for origin of paths
clcolors<-rainbow(round(nc*1.05))[1:nc]
cellcolors<-rep("",ncell)
for (i in 1:nc) cellcolors[apres@clusters[[i]]]<-clcolors[i]




NCORES <- 12
register(bpstart(MulticoreParam(workers=NCORES)))

#preprocessing from counts
E<-C[,-(1:nb[1])] #only MFRP cells
E<-E[rowSums(E)>0,]
dim(E)
[1] 17893  2059

library(mygene)
ens<-rownames(E)
q<-queryMany(ens,scopes="ensembl.gene")
good<-!duplicated(q$query)
uq<-q[good,c(1,4,6,5)]
sym<-uq$symbol
gene<-uq$entrezgene

rownames(E)<-sym




#create SummarizedExperiment object
metadata<-data.frame("MFRP_cluster"=MFRP_cluster)
#make se object
se <- SummarizedExperiment(assays = list(counts = E),colData =
```

```
metadata)

#drop cells with not enough alignment % of housekeeping genes
data(housekeeping) #load HUMAN housekeeping symbols
hk = rownames(se)[toupper(rownames(se)) %in% housekeeping$V1]
#this is 490 genes
#keep only cells with solid expression of housekeeping genes
mfilt <- metric_sample_filter(assay(se),pos_controls = rownames(se)
%in% hk,hard_nreads=1500,zcut =3, mixture =FALSE,plot =TRUE)
#simplify to single logical, use only list components that are defined
mfilt<-!apply(simplify2array(mfilt[!is.na(mfilt)]), 1, any)
mean(mfilt)
[1] 0.7901894
se <- se[, mfilt]
dim(se)
[1] 17893  1627
se_MFRP_cluster<-MFRP_cluster[mfilt] #MFRP cluster membership of cells
that pass filter
se_cellcolors<-cellcolors[mfilt] #remember their MFRP cluster colors
se_ncell<-sum(mfilt) #this many cells are in se object


#take the "core" set of genes with greatest variance
vars <- rowVars(log1p(assay(se))) #this is natural log(x+1)
names(vars) <- rownames(se)
vars <- sort(vars, decreasing =TRUE)
core <- se[names(vars)[1:1000],] #take top 1000 most variable genes

#-1000/dim(E)[1]
#this is at 94.4%-ile of variance

#dimensionality reduction using the zero-inflated negative binomial-
based wanted variation extrac-tion (ZINB-WaVE) method implemented in
the Bioconductor R package zinbwave.
system.time(se2<-
zinbwave(core,K=4,residuals=TRUE,normalizedValues=TRUE))
#   user   system  elapsed
#325.744   37.024 1412.846

norm <- assays(se2)$normalizedValues
norm[1:3,1:3]

#this is a check if the MFRP clusters make sense
pca <- prcomp(t(norm))
set.seed(2357)
o<-sample(se_ncell)
plot(pca$x[o,],col=se_cellcolors[o],pch=20,main="") #draw cells in
original MFRP colors in random order
#yes this looks OK
```

```
#the zinbwave with K=2 looks better
se3 <- zinbwave(core, K =2, residuals =TRUE,normalizedValues =TRUE)
#this takes a while
X<-se3@int_colData@listData$reducedDims[[1]] #reduced dimensions in
zinbwe
plot(X[o,],col=se_cellcolors[o],pch=20,main="") #draw cells in
original MFRP colors in random order

#do a MDS with Euclidean scaling using the K=50 representation:
W <- as.matrix(se2@reducedDims[[1]])
d <- dist(W)
fit <- cmdscale(d, eig =TRUE, k =2)
plot(fit$points, col = se_cellcolors, main ="",pch =20, xlab
="Component 1", ylab ="Component 2")
legend(x ="topright", legend = 1:nc, cex = .8,pch=20,col=clcolors,
title ="Cluster")

#################this did not work
well##########################################
#do RSEC clustering on the W 4-dim matrix
#use the resampling-based sequential ensemble clustering (RSEC)
framework implemented in the RSECfunction from the Bioconductor R
package clusterExperiment.
#As  in  supervised  learning,  resampling  greatly  improves  the
stability  of  clusters
seObj <- SummarizedExperiment(t(W), colData = colData(core))
print(system.time(ceObj<-
RSEC(seObj,k0s=4:12,alphas=c(0.1),betas=0.8,reduceMethod="none",cluste
rFunction="hierarchical01",minSizes=3,ncores=NCORES,isCount=FALSE,dend
roReduce="none",dendroNDims=NA,subsampleArgs=list(resamp.num=100,clust
erFunction="kmeans",clusterArgs=list(nstart=10)),verbose=TRUE,consensu
sProportion=0.7,mergeMethod="locfdr",random.seed=2357,consensusMinSize
=10)))
    user   system  elapsed
1398.008   49.171  289.096
#if K is set high, like 50, it will take a long time to finish
save(ceObj,file="ceObj.RData")
plotClusters(ceObj, colPalette = c(bigPalette, rainbow(199))) #looks
like I need to merge clusters
############################################################




#######################begin Slingshot plotting
work######################################
#since RSEC makes a lot of clusters, let's use the original cluster
ID's for lineage tracking
#Now Slingshot
#take cluster 8 as the origin of lineage
```

```
X2<-se2@reducedDims[[1]] #this is the 4-dim zinbwave representation
lineages <- getLineages(X2, clusterLabels = se_MFRP_cluster,
start.clus ="8")
pairs(lineages, type="lineages",col=se_cellcolors)
lineages <- getCurves(lineages)
pdf("lineages.pdf",width=20,height=20)
plot(X2[,1:2],col=se_cellcolors,pch=20,cex=2)
lines(lineages)

pairs(lineages, type="curves",col=se_cellcolors)
pairs(lineages, type="curves",horInd=2,verInd=1,col=se_cellcolors)
dev.off()
#special panel for Shyamanga
pdf("lineages_1_2.pdf")

plot(X2[,1:2],col=se_cellcolors,pch=20,cex=1.5,xlim=c(-5,2),ylim=c(-3,
3))
#  lines(lineages@curves[[1]]$s[,2:1])
   lines(lineages)
   legend(x ="topright", legend = 1:nc, cex = 1.,pch=20,col=clcolors,
title ="Cluster")
dev.off()
lineages
#class: SlingshotDataSet

# Samples Dimensions
#    1627          4

#lineages: 4
#Lineage1: 8  2  5  4  10  16  12
#Lineage2: 8  2  9  1  14  13
#Lineage3: 8  2  5  6  15
#Lineage4: 8  2  7

#curves: 4
#Curve1: Length: 9.7525 Samples: 1209.57
#Curve2: Length: 7.7882 Samples: 1113.64
#Curve3: Length: 4.7581 Samples: 1270.64
#Curve4: Length: 5.08   Samples: 1052.6

nlin<-length(lineages@lineages)
for (i in 1:nlin) {
icurve<-i
t <- slingPseudotime(lineages)[,icurve]
y <- assays(se2)$normalizedValues
gam.pval <- apply(y,1,function(z){
  d <- data.frame(z=z, t=t)
  tmp <- gam(z ~lo(t), data=d)
  p <- summary(tmp)[4][[1]][1,5]
})
```

```
topgenes <- names(sort(gam.pval))[1:100]
heatdata <- y[rownames(se2) %in% topgenes, order(t, na.last =NA)]
heatclus <- se_MFRP_cluster[order(t, na.last =NA)]
ce <- ClusterExperiment(heatdata, heatclus, transformation = identity)
#match to existing
cols <- clusterLegend(ceObj)$combineMany[, "color"]
names(cols) <- clusterLegend(ceObj)$combineMany[, "name"]
ce@clusterLegend[[1]][,"color"]<-
clcolors[as.numeric( ce@clusterLegend[[1]][,"name"] )]

pdf(paste0("lineage ",i,".pdf"))
   plotHeatmap(ce, clusterSamplesData =c("orderSamplesValue"), breaks
= .99,colorScale=seqPal2)
dev.off()
}
```

```
#make ultimate heatmap for lineage No. 3 per Shyamanga's request
cellClusterLabels<-lineages@clusterLabels #matrix 0/1 with colnames as
clusters and rownames as cell ID:
colnames(cellClusterLabels)
# [1] "5"  "2"  "4"  "9"  "6"  "8"  "10" "15" "16" "13" "12" "1"  "14"
"7"
icurve<-3
icurveClusters<-lineages@lineages[[icurve]] #a vector of clusters
along the lineage trajectory
#[1] "8"  "2"  "5"  "6"  "15"
icurveCellClusterLabels<-cellClusterLabels[,icurveClusters] #columns
are "8"  "2"  "5"  "6"  "15", some rows have all 0's
cell.in.lineage<-apply(icurveCellClusterLabels>0,1,any) #1246 cells


t <- slingPseudotime(lineages)[,icurve] #contains NA for cells that
are not in the lineage #icurve
y <- assays(se2)$normalizedValues
gam.pval <- apply(y,1,function(z){
  d <- data.frame(z=z, t=t)
  tmp <- gam(z ~lo(t), data=d)
  p <- summary(tmp)[4][[1]][1,5]
})
topgenes <- names(sort(gam.pval))[1:100]
#heatdata <- y[rownames(se2) %in% topgenes, order(t, na.last =NA)]
```

```
#the order by t mixes up cluster labels a bit, so let's enforce
cluster label first, in order of icurveClusters, then t
#first convert se_MFRP_cluster such that clusters 8,2,5,6,15 become
A,B,C,D,E and the rest of them are NA
Labels<-LETTERS[1:length(icurveClusters)]
names(Labels)<-icurveClusters
all.cell.Labels<-Labels[as.character(se_MFRP_cluster)]
cell.order<-order(all.cell.Labels,t,na.last=NA) #t breaks the ties in
all.cell.Labels, NA's anywhere are tossed
#works like a charm, but it isn't the right thing to do

cell.order<-order(t,all.cell.Labels,na.last=NA) #this sorts by t and
keeps only clusters 8,2,5,6,15

cell.cluster.matrix<-cellClusterLabels[cell.order,icurveClusters]

heatdata <- y[rownames(se2) %in% topgenes,cell.order]

#now sort the genes as I like them
#d<-dist(heatdata) #100x100 distance object
#the problem is, dist is calculated from super noisy data.
library(caTools)
y<-t(runmean(t(heatdata),k=100))
d<-dist(y)
h<-hclust.olo(d,method="ward.D")
h.heatdata<-heatdata[h$order,]

ce <- ClusterExperiment(h.heatdata,cell.cluster.matrix, transformation
= identity)
for (cl in 1:length(icurveClusters)) ce@clusterLegend[[cl]]
[,"color"]<-c("grey95",clcolors[as.numeric(icurveClusters[cl])])
save(ce,file="ce.RData")

pdf(paste0("lineage ",i,".pdf"))
#the list(...) makes sure that no clustering on the features (rows)
will be done!!!!!!!!!!!!!!!!!
plotHeatmap(ce,clusterSamplesData="orderSamplesValue",breaks=.97,color
Scale=seqPal2,clusterFeaturesData=list(1:100),treeheight=0,annLegend=F
ALSE,whichClusters="all")

dev.off()
```

```
#this was from vignette:
data('slingshotExample')
condition <- factor(rep(c('A','B'), length.out = nrow(rd)))
condition[110:140] <- 'A'

sds <- slingshot(rd, cl)
#sds@curves$curve1
#sds@curves$curve2
plot(rd, col = cl, asp = 1)
lines(sds, type ='c', lwd = 3)
```
